# Dual-Functional Amine-Modified Aluminum-Doped MCM-41 Nanoparticles for Concurrent Zoledronic Acid Adsorption and Geranylgeraniol Delivery for Prevention of Medication-Related Osteonecrosis of the Jaw

**DOI:** 10.1021/acsmaterialsau.5c00112

**Published:** 2025-10-13

**Authors:** Pornchanok Pichaipanich, Weerachai Singhatanadgit, Boonlom Thavornyutikarn, Piyarat Sungkhaphan, Setthawut Kitpakornsanti, Soraya Pornsuwan, Wanida Janvikul

**Affiliations:** † National Metal and Materials Technology Center, 61191 National Science and Technology Development Agency, Pathum-thani 12120, Thailand; ‡ Faculty of Dentistry and Research Unit in Mineralized Tissue Reconstruction, Thammasat University (Rangsit Campus), Pathum-thani 12121, Thailand; § Faculty of Science, Mahidol University, Bangkok 10400, Thailand

**Keywords:** medication-related osteonecrosis of the jaw, zoledronic acid, geranylgeraniol, aluminum-doped mesoporous silica nanoparticle, amine functionalization, drug delivery and adsorption

## Abstract

This study aimed to develop a bifunctional nanomaterial that could simultaneously adsorb zoledronic acid (ZA) and release geranylgeraniol (GGOH) to reverse ZA-induced cytotoxicity. The synthesized aluminum-doped mesoporous silica nanomaterial (AM) was subsequently amine-functionalized by 3-aminopropyltriethoxysilane, generating both amine- and aluminum-containing nanomaterial (NAM), to enhance the ability of nanoparticles to adsorb GGOH. The comprehensive characterization results confirmed the successful aluminum-doping and amine-functionalization of the nanoparticles. The results acquired from both thermogravimetric analysis and high-performance liquid chromatography demonstrated that NAM, rather than AM, served as a good nanocarrier for GGOH loading and controlled-releasing. NAM exhibited up to 12.48% GGOH loading efficiency and GGOH sustained release for over 10 days with a release profile best fitted by the Higuchi model (*R* ^2^ = 0.9868), indicating a diffusion-controlled mechanism. Although AM demonstrated much higher ZA adsorption (>95%), NAM still retained moderate ZA adsorption (∼30%). In vitro assays using RAW 264.7 murine cells revealed that GGOH-loaded NAM was noncytotoxic and completely reversed ZA-induced cytotoxicity and metabolic impairment. Furthermore, it displayed negligible hemolytic activity (<0.5%). The combination of targeted drug delivery and bisphosphonate sequestration via nanostructured silica nanocarriers presents a promising therapeutic approach with translational potential in the prevention of medication-related osteonecrosis of the jaw. The promising cellular results, serving as a preclinical foundation, provide a stepping stone toward in vivo applications.

## Graphical abstract

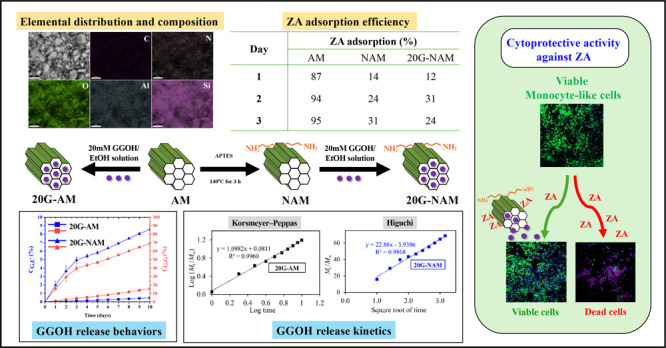

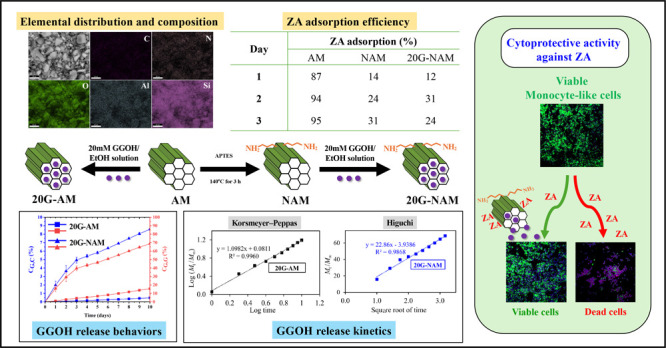

## 1 Introduction

The prolonged use of high-dose antiresorptive medications, such as zoledronic acid (ZA), can lead to a serious adverse effect known as medication-related osteonecrosis of the jaw (MRONJ), which is characterized by pain and exposed necrotic jawbone.^1^ Due to the absence of established clinical practice guidelines for MRONJ management, standard supportive care is currently recommended.^1^ The treatment incurs a cost of up to $20,000 per individual, with an estimated 12-month post-treatment observation period, and a significant proportion of patients, approximately 25%, continue to exhibit symptoms following treatment.^2^ Preventive strategies to mitigate the risk of MRONJ remain a challenge.

Potential cytotoxicity of ZA can decrease the number of viable osteoclast precursors and mesenchymal stem cells (MSCs), thereby reducing the number of functionally active osteoclasts and osteoblasts, respectively, and inducing bone necrosis after jawbone injury.^1,3^ ZA binds strongly to hydroxyapatite (HA) in bone and can be released from HA molecules to be free (unbound) ZA in response to the acidic microenvironment by fully functional osteoclasts during bone resorption. These free ZA molecules can be taken up by bone cells and inhibit the biosynthesis of geranylgeranyl pyrophosphate (GGPP), which is important for osteoclasts’ viability and function.^1^ Molecules with high ZA binding affinity can reduce the level of free ZA available for cellular uptake.^4–6^ We have previously demonstrated that nanocomposite hydrogels releasing geranylgeraniol (GGOH), which is intracellularly converted to GGPP, can reverse ZA cytotoxicity at both cellular and molecular levels.^7–9^ These studies reported GGOH delivery platforms, using amine-functionalized MCM-41/carboxymethyl chitosan composite hydrogels for cytoprotection against ZA.^8,9^ Their primary function was to release GGOH via diffusion from the GGOH-loaded-composite hydrogels through the nanoparticle pores and hydrogel matrix in a controlled and sustained manner, thereby achieving the therapeutic effect. However, GGOH released from these hydrogels could be toxic to osteoclast precursors while preventing ZA-induced cytotoxicity to MSCs.^9^ Additionally, GGOH may not fully mitigate the cytotoxicity induced by a high dose of ZA.^7,10^ It was suggested that the protective effect of GGOH against ZA was dose-dependent, only within an optimal concentration range and directly proportional to the ZA concentration, with the optimal GGOH:ZA ratio not exceeding 100% to avoid toxicity.^7,10,11^ For the prevention of MRONJ, sequestrating ZA is therefore critical to reduce its bioavailability below a level where its adverse effects can be rescued by a nontoxic concentration of GGOH. This dual functionality may be achieved by using a tunable nanocarrier platform for both ZA adsorption and GGOH delivery, thus enhancing the protective index of cellular toxicity induced by high-dose ZA.

Biocompatible adsorbents, such as calcium phosphates (e.g., hydroxyapatite, β-tricalcium phosphate, and biphasic calcium phosphate), can bind to free ZA, reducing its bioavailability, minimizing cellular uptake, and thereby lessening the risk of developing MRONJ in animal models.^5,6,12^ While these materials have shown promise in animal models, their surface structure and varying ZA adsorption capacities may hinder their effectiveness. Developing novel biomaterials with enhanced ZA adsorption properties could offer a more effective solution. For instance, porous activated carbon incorporated with magnesium oxide, with the ability to adsorb ZA in vitro*,* has been recently prepared.^4^ However, the reversal effect against ZA cytotoxicity has not yet been determined.

Mesoporous silica Mobil Composition of Matter No. 41 (MCM-41), characterized by a highly ordered hexagonal arrangement of cylindrical pores, offers significant potential for drug delivery applications.^13^ Its large specific surface area and porosity readily facilitate the adsorption of drug molecules not only at the core of MCM-41 pores but also on the external surface of the material.^14^ Nonetheless, its hydrophilic surfaces consisting of free silanol groups can limit the adsorption of some hydrophobic drugs and active agents. To address this issue, our previous study has recently reported that the surface functionalization of MCM-41 using 3-aminopropyltriethoxysilane (APTES) enhanced the capacity of the resulting nanomaterial, i.e., amine-containing MCM-41 (N-MCM-41), to load and sustainably release poorly water-soluble GGOH.^15^ In addition, amine-functionalization of MCM-41 is reported to be an effective strategy to create a material with a high alendronate loading capacity.^16^ Meanwhile, to enhance the binding affinity of ZA to MCM-41 nanoparticles, the surface modification of the nanomaterial with metal cations is a promising approach as it leverages the established ability of metal cations to effectively adsorb bisphosphonates that possess two phosphate groups linked by a two- or three-carbon chain.^17–19^ For instance, trivalent aluminum cations (Al^3+^) have been demonstrated to form equimolar monomeric complexes with bisphosphonates, suggesting its bisphosphonate chelating activity.^17^ Moreover, low concentrations of aluminum ions have been shown to stimulate osteoblast proliferation and differentiation.^20^ Alginate films cross-linked with aluminum ions exhibit increased adsorption of serum proteins.^21^

Aluminum-containing MCM-41 (Al-MCM-41) nanoparticles have been applied in various research fields due to the tetrahedrally coordinated aluminum atoms that can create active sites for catalysis, adsorption, and controlled drug delivery.^22,23^ Studies have demonstrated that incorporating aluminum into the MCM-41 framework significantly influenced its drug delivery properties. It increased the number of acid sites, creating stronger interactions with drug molecules. For instance, it led to a slower and more sustained release of ibuprofen compared to nonaluminated MCM-41.^24^ Al-modified MCM-41 has also been successfully used as a drug carrier for other therapeutics, such as mesalamine^25^ and levofloxacin.^26^ The aluminum content directly affected the drug release profile in these systems. For example, the usage of Al-MCM-41 nanoparticles as a carrier for the antibiotic oxytetracycline showed that a higher aluminum content and increased antibiotic loading generally led to a slower drug release rate.^27^ Herein, we report, for the first time, the development of dual-functional modified Al-MCM-41 nanoparticles that serve as a potential biomaterial to prevent MRONJ associated with ZA. The Al-MCM-41 nanomaterial was initially amine-functionalized with APTES, generating both amine- and aluminum-containing nanomaterial (N–Al-MCM-41) that was simultaneously employed for GGOH loading and release as well as ZA adsorption. The properties, GGOH-loading/releasing capacity, and ZA adsorption efficiency of the resulting NH_2_-modified Al-doped-MCM-41 nanoparticles were comprehensively analyzed, in comparison with those of the starting Al-MCM-41 nanomaterial. In addition, the in vitro cytotoxicity, reversal activity against ZA, and hemolysis of the GGOH-loaded–N-Al-MCM-41 nanomaterial were also investigated.

## 2 Materials and Experiments

### 2.1 Materials

Cetyltrimethylammonium bromide (CTAB) (MW = 364.45 g/mol), sodium hydroxide (NaOH) (MW = 40 g/mol), sodium aluminate (NaAlO_2_) (MW = 81.97 g/mol), tetraethyl orthosilicate (TEOS) (MW = 208.33 g/mol), 3-aminopropyltriethoxylsilane (APTES) (MW = 221.37 g/mol), and geranylgeraniol (GGOH) (MW = 290.48 g/mol) were supplied by Sigma-Aldrich Corporation. Zoledronic acid monohydrate (ZA) (MW = 290.11 g/mol) was purchased from Tokyo Chemical Industry (TCI) Co., Ltd. Absolute ethanol (EtOH) (AR grade, 99.9% denatured) was bought from QRëC Chemical Co., Ltd. Toluene (AR grade, 99.9%) was purchased from Merck KGaA. Methanol (MeOH) (HPLC grade, ≥99.9%) were obtained from Thermo Fisher Scientific. Acetonitrile (ACN) (HPLC grade, 99.9%) was purchased from Sigma-Aldrich Corporation. All reagent-grade chemicals were used as received without further purification.

### 2.2 Synthesis of Aluminum-Containing Mesoporous Silica Nanoparticles

The aluminum-containing mesoporous silica material (Al-MCM-41 or shortly coded as AM) was synthesized using a modified procedure previously reported by Cai et al.^28^ As briefly illustrated in Scheme 1, 1.0 g of CTAB was initially added to an aqueous solution of 3.5 mL of 2 M NaOH in 480 mL of distilled water. The mixture was subsequently heated at 80 °C and stirred for 15 min at 625 rpm. Once CTAB was completely dissolved, 0.196 g of NaAlO_2_ and 4.8 g of TEOS were consecutively added under vigorous stirring, yielding a white slurry. The reaction was continuously stirred at 80 °C for 2 h. Next, the resulting white powder was filtered, rinsed with distilled water, and dried at room temperature. The powder was ultimately calcined at 560 °C for 5 h with a ramp rate of 1 °C/min.

**Scheme 1:**
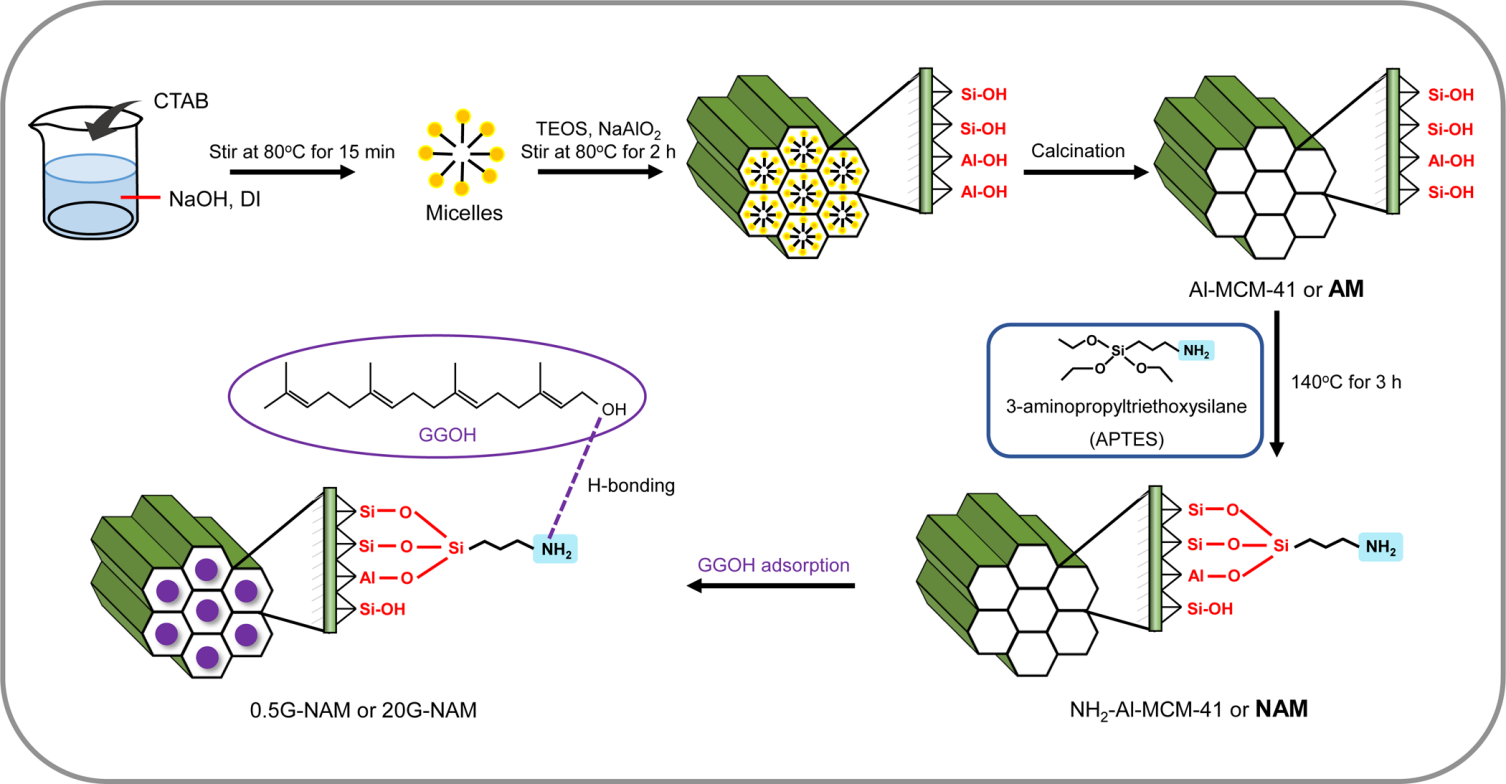
Schematic Illustration of the Preparation of Geranylgeraniol-Loaded Amine- and Aluminum-Containing Mesoporous Silica Nanoparticles

### 2.3 Amine-Functionalization of Aluminum-Containing Mesoporous Silica Nanoparticles

As shown in Scheme 1, to enhance the surface hydrophobicity of the mesoporous silica nanomaterial for adsorption of GGOH, Al-MCM-41 was functionalized by APTES using a modified method previously reported.^29^ Briefly, 1 g of Al-MCM-41 was primarily dried in an oven at 80 °C overnight. The dried Al-MCM-41 was subsequently suspended in dry toluene (29.5 mL) under a N_2_ atmosphere at 90 °C for 10 min. Then, 0.5 mL of APTES was added dropwise into the slurry under stirring before the whole mixture was heated at 140 °C for 3 h. After cooling to room temperature, the resulting amine-functionalized aluminum-containing mesoporous silica nanomaterial (NH_2_–Al-MCM-41 or concisely coded as NAM) was recovered by centrifugation, washed vigorously with a 50:50 (v/v) mixture of distilled water and methanol, and then dried in a vacuum oven at room temperature for 12 h.

### 2.4 Characterization of Al-MCM-41 and NH_2_–Al-MCM-41 Nanoparticles

An X-ray powder diffractometer (TTRAX III, RIGAKU, Japan), equipped with a copper radiation source (CuKα, λ = 0.154 nm), was employed to obtain the XRD patterns of both AM and NAM. The XRD analysis was performed using the following conditions: 50 kV, 300 mA, angular range (2θ) of 1°–10°, step size of 0.02°, and scanning rate of 2°/min. The specific surface area, total mesopore volume, and pore size of each nanomaterial were examined by the nitrogen adsorption–desorption isotherms using a Brunauer–Emmett–Teller (BET) surface area analyzer (Micromeritics Instruments, USA). Data were processed with 3Flex version 5.02 software (Micromeritics, USA). The pore size distribution of each nanocarrier was calculated by the Barrett–Joyner–Halenda (BJH) method. The particle size distribution and size averages of each nanomaterial were also determined using dynamic light scattering (DLS) measurement with Zetasizer Nano series ZS (Malvern, UK). The particle suspension in deionized (DI) water was sonicated prior to the analysis. The elemental composition and atomic percentages of N and Al of the NH_2_–Al-MCM-41 nanomaterial were determined using a scanning electron microscope equipped with energy dispersive X-ray analysis (SEM-EDX) (S-3400N, Hitachi, Japan); the gold-coated specimens were examined using an accelerating voltage of 20 kV.

### 2.5 Loading of GGOH into Mesoporous Silica Nanoparticles

In this study, GGOH (coded as G), a poorly water-soluble diterpenoid alcohol, was loaded into both Al-MCM-41 (AM) and NH_2_–Al-MCM-41 (NAM). As concisely demonstrated in Scheme 1, 20 mg of each dried mesoporous silica nanomaterial was placed in 1 mL of a 0.5 or 20 mM GGOH/EtOH solution. All slurries were individually vortexed for 2 min and then sonicated for 15 min at room temperature. The entire mixtures were next shaken at 130 rpm for 2 days in a shaking incubator at room temperature. To enhance the GGOH loading efficiency in the nanoparticles, during the 2 days of shaking, the mixtures were transferred three times a day into a vacuum oven, where they were exposed to a negative pressure of −30 inHg for 2 min. Afterward, the whole slurries were individually centrifuged to separate out the different GGOH-loaded nanoparticles, coded as 20G-AM, 0.5G-NAM, and 20G-NAM, from the supernatants. The collected GGOH-loaded specimens were dried in a vacuum oven at −30 inHg for 24 h prior to usage.

### 2.6 Quantification of GGOH in Mesoporous Silica Nanoparticles

The amounts of GGOH actually present in the individual nanocarriers were quantified by thermogravimetric analysis (TGA) (TGA 2 STARe System, Mettler Toledo, USA). The TGA thermograms were collected over a temperature range of 30 to 800 °C, while the specimens were heated at a rate of 10 °C/min under a nitrogen environment from 30 to 600 °C and an oxygen environment from 600 °C onward. To determine the GGOH loading and releasing efficiencies, the specimens of each GGOH-carrying nanomaterial before and after GGOH releasing process were successively brought into TGA analysis. The weight percentages of GGOH actually loaded (*T*_G,TGA_) in the nanocarrier and left (*L*_G,TGA_) in the nanocarrier after the releasing process were directly calculated from the data obtained in the TGA thermograms where the combustion of GGOH was characteristically observed in the temperature range of 150–350 °C in this study. The weight percentage of GGOH totally released from the carrier (*R*_G,TGA_) was subsequently determined by subtracting *L*_G,TGA_ from *T*_G,TGA_.

### 2.7 In Vitro GGOH Release Behaviors

The amount of GGOH liberated from each GGOH-carrying nanomaterial was measured by high-performance liquid chromatography (HPLC, 1260 Infinity, Agilent Technologies, USA). The HPLC analysis was performed by using a C-18 column (150 × 4.6 mm, 5 μm, TSKgelODS-100 V) as a stationary phase and a mixed solvent (90:10, v/v) of acetonitrile solution and double DI water as a mobile phase with a flow rate of 0.5 mL/min. In brief, 20 mg of each GGOH-loaded sample (*n* = 2) was immersed in 1.2 mL of a releasing medium containing phosphate buffered saline (PBS) and 10% fetal bovine serum (FBS) for 10 days at 37 °C. The fresh medium (1.2 mL) was regularly added to maintain the volume of solution constant after daily supernatant collection. Prior to HPLC injections, the collected liquids were individually mixed with ethanol, frozen at −80 °C for 1 day, centrifuged to precipitate FBS, and then filtered using 0.45 μm PTFE membranes. The injection volume of HPLC sample was 100 μL, and the detection wavelength was set at 210 nm. The amount of GGOH in each withdrawn release medium was quantified by correlating a peak area in an HPLC chromatogram to the GGOH standard curve constructed using known GGOH concentrations prepared by the protocol described above.

The percentage of GGOH totally released per weight of a GGOH-loaded carrier (*R*_G,HPLC_), the cumulative GGOH release per weight of a GGOH-loaded carrier (*C*_G,C_), the cumulative GGOH release per total amount of GGOH actually loaded (*C*_G,G_), and the percentage of GGOH remaining per weight of a GGOH-loaded carrier (*L*_G,HPLC_) were calculated using the following eqs 1–4, respectively.

$$R_{G,\mathrm{HPLC}}(\%)=(R_{T}/W_{i})\times 100$$ 1

where *R*_T_ represents the total amount (μg) of GGOH released ( $\sum_{t=0}^{t=t}R_{t}$ ) measured by HPLC when *t* is a releasing time (day) (*t* = 1–10). *W_i_* represents the initial dry weight (μg) of the GGOH-loaded carrier (*W_i_* = 20,000 μg).

$$\mathrm{Cumulative}\;\mathrm{release}\;(C_{G,C})\;(\%)=\sum (R_{t}/W_{i})\times 100$$ 2

$$\mathrm{Cumulative}\;\mathrm{release}\;(C_{G,G})\;(\%)=\sum (R_{t}/W_{d})\times 100$$ 3

where *R_t_* and *W_d_* represent the amount (μg) of GGOH released at time *t*, analyzed by HPLC, and the total amount (μg) of GGOH actually loaded, determined by TGA, respectively.

$$L_{G,\mathrm{HPLC}}\;(\%)=T_{G,\mathrm{TGA}}-R_{G,\mathrm{HPLC}}$$ 4

where *T*_G,TGA_ represents the percentage of GGOH loaded per weight of a GGOH-loaded carrier before the release study, measured by TGA.

### 2.8 In Vitro GGOH Release Kinetics

To investigate the release kinetics of GGOH from the nanoparticles, the individual release profiles obtained from the HPLC analysis were fitted with the following mathematical models: First-order, Higuchi, Korsmeyer–Peppas, and Hixson–Crowell models using the eqs 5–8 below. The fitting or linear regression plots were generated using Microsoft Excel (Microsoft Office Professional Plus 2021).

$$\mathrm{First}-\mathrm{order}\;\mathrm{equation}\;\log\;C=\log\;C_{0}-K_{1}t/2.303$$ 5

where *C* is the percentage of remaining GGOH at time (day) *t*; *C*_0_ is the total amount of GGOH actually loaded, *K*_1_ is a first-order rate constant, and *t* is a release time (day).^30^

$$\mathrm{Higuchi}\;\mathrm{equation}\;M_{t}/M_{\infty }=K_{H}(t{)}^{1/2}$$ 6

where *M*_*t*_ and *M*_∞_ are the percentages of cumulative GGOH release at time (day) *t* and infinite time (day) (*M*_∞_ = 100%), respectively, per total amount of GGOH actually loaded; *K*_H_ is a Higuchi dissolution constant, and *t* is a release time (day).^31^

$$\mathrm{Korsmeyer}-\mathrm{Peppas}\;\mathrm{equation}\;M_{t}/M_{\infty }=kt^{n}$$ 7

where *M* *_t_* and *M*_∞_ are the percentages of cumulative GGOH release at time (day) *t* and infinite time (day) (*M*_∞_ = 100%), respectively, per total amount of GGOH actually loaded; *k* is a release kinetic constant; *t* is a release time (day), and *n* is a release exponent which indicates the GGOH release mechanism.^31^

$$\mathrm{Hixson}-\mathrm{Crowell}\;\mathrm{equation}\;(M_{0}{)}^{1/3}-(M_{t}{)}^{1/3}=\kappa t$$ 8

where *M*_0_ represents the initial amount of GGOH actually loaded in the nanoparticle (*M*_0_ = 100%); *M*_*t*_ is the percentage of cumulative GGOH release per total amount of GGOH actually loaded at time (day) *t*; κ is a constant incorporating the surface volume relation, and *t* is a release time (day).^9^

### 2.9 ZA Adsorption Efficiency of Mesoporous Silica Nanoparticles

To assess the ability of all types of nanoparticles to adsorb zoledronic acid (ZA), 0.25 mL of each aqueous suspension of nanoparticles in DI water (200 μg/mL) was mixed with 0.25 mL of 200 μM ZA aqueous solution in an Eppendorf tube. Independent sets of each mixture were shaken at 100 rpm at 37 °C for 3 days. On each day, the individual suspensions (*n* = 2) were centrifuged, and their supernatants were collected and then subjected to a filtration through 0.45 μm PTFE membranes before being injected into HPLC using a C-18 column (250 × 3.0 mm, 5 μm, Inertsil ODS-3) as a stationary phase and a mixed solvent (90:10, v/v) of phosphate buffer (pH 2.6) and methanol as a mobile phase with a flow rate of 0.3 mL/min. The injection volume of HPLC sample was 50 μL, and the ZA detection was set at a wavelength of 210 nm. The efficiency of ZA adsorption of each nanomaterial was determined using the eq 9. To construct the calibration curve for ZA, a 17 mM stock solution was initially prepared by dissolving 5 mg of ZA in 1 mL of deionized water. This stock was then diluted to a 1 mM intermediate solution from which working standard solutions of 100, 80, 50, 20, and 10 μM were prepared. These standard solutions were subsequently analyzed by HPLC to generate the calibration curve.

$$\mathrm{ZA}\;\mathrm{adsorption}\;(\%)=\left[\left(A_{0}-A_{t}\right)/A_{0}\right]\times 100$$ 9

where *A*_0_ and *A*_*t*_ represent the amount (mg) of ZA initially used and the remaining amount (mg) of ZA after the immersing time (day) *t*, respectively.

### 2.10 In Vitro Cytocompatibility and Cytoprotective Activity against ZA of GGOH-Loaded Nanoparticles

RAW 264.7 murine monocyte-like cells (RAW cells; ATCC) were cultured in Dulbecco’s Modified Eagle’s Medium (DMEM; Gibco Life Technologies Ltd., Paisley, UK) supplemented with 10% fetal bovine serum (FBS), 200 U/mL penicillin, 200 μg/mL streptomycin, and 2 mM l-glutamine (all from Gibco) at 37 °C in a humidified atmosphere containing 5% CO_2_. Passages 15–20 were used for all experiments. The 20G-NAM nanoparticles were sterilized by UV irradiation for 1 h prior to cell treatment.

Cells were seeded at a density of 3 × 10^4^ cells/cm^2^ in 24-well plates and cultured for 18 h. Subsequently, cells were exposed to 50 μM ZA (Aclasta, Novartis Pharmaceuticals UK Ltd., UK) and the nanoparticles at concentrations of 0, 100, 200, and 300 μg/mL for 72 h. The culture medium (0.3 mL/well) was replenished every 24 h with a fresh medium containing the respective treatments. Cells cultured without ZA and GGOH-loaded nanoparticles served as controls for cytocompatibility assessment. Cells treated with ZA alone were used as controls to evaluate the cytoprotective effect of the GGOH-loaded nanoparticles against ZA-induced toxicity.

Confocal laser scanning microscopy of live and dead cell staining and resazurin assay were used to determine cell viability and metabolic activity of RAW cells, respectively. For live/dead cell staining, samples were incubated with 25 μM carboxyfluorescein diacetate succinimidyl ester (CFSE) in PBS for 3 min at 4 °C, followed by two washes with PBS. Subsequently, samples were incubated with 2 μg/mL propidium iodide (PI) in PBS for 15 min at room temperature and washed twice with PBS. Cells were fixed with 4% paraformaldehyde for 15 min at room temperature and then stained with 1 mg/mL 4′,6-diamidino-2-phenylindole (DAPI) for 15 min at room temperature. Stained samples were analyzed under a confocal fluorescence microscope (Nikon Ti Eclipse, Nikon Instruments Inc., NY, USA). Four fields of view per well were imaged, and experiments were performed in duplicate, with consistent results obtained from two independent experiments. For the resazurin assay, 500 μL of a 0.5 mM resazurin dye solution was added to each well of a 24-well plate and incubated for 2 h at 37 °C in the dark. Subsequently, the solution was centrifuged at 1000 *g* for 5 min, and 200 μL of the supernatant was transferred into a 96-well plate. Fluorescence measurements were conducted using a fluorometer (Thermo Scientific Varioskan Flash Spectral Scanning Multimode Reader) with excitation at 530 nm and emission at 590 nm. Data are presented as the mean ± standard deviation (SD) of four replicates from two independent experiments with consistent results.

For the apoptosis assay, cells were seeded and treated as described above. The 20G-NAM nanoparticles were used at concentrations of 0, 100, and 200 μg/mL for 24 h. Following treatment, cells were extensively washed with cold PBS and harvested. The cells were then washed twice with cold annexin V binding buffer containing 10 mM HEPES/NaOH, pH 7.4, 140 mM NaCl, and 2.5 mM CaCl_2_ and stained with Annexin V-FITC kit according to the manufacturer’s instructions (Miltenyi Biotech, CA, USA). Samples were analyzed by flow cytometry (CytoFLEX Flow Cytometer and CytExpert software, Beckman Coulter, CA, USA). A total of 20,000 events were acquired per sample. Quadrant gating distinguished the following populations: annexin V^–^/PI^–^ viable cells, annexin V^+^/PI^–^ early apoptotic cells, annexin V^+^/PI^+^ late apoptotic cells, and annexin V^–^/PI^+^ necrotic cells. Data are expressed as the percentage of cells in each quadrant.

### 2.11 Assessment of the Hemolytic Index of GGOH-Loaded Nanoparticles

The hemolytic activity of 20G-NAM nanoparticles was assessed using a biologically relevant 3D clotted human whole-blood model, modified from a previously reported protocol.^32^ Fresh whole blood samples were collected from two healthy volunteers. Participants provided informed consent to the use of their blood, in accordance with the protocol approved by the Ethics Review Sub-Committee for Research Involving Human Research Subjects of Thammasat University No. 3 (120/2566) and the Institutional Biosafety Committee Thammasat University (012/2567). A stock solution of 20G-NAM was prepared at 20 mg/mL in 0.9% NaCl solution under ultrasonication for 5 min before being mixed with fresh whole blood to achieve the indicated final nanoparticle concentrations.

Aliquots of 250 μL of whole blood mixed with 20G-NAM at 0, 250, 500, 1000, and 2000 μg/mL were incubated for 1 h at 37 °C to allow blood clot formation. Subsequently, 250 μL of PBS was added to each clotted blood sample, followed by an additional 2-h incubation at 37 °C. The samples were then centrifuged at 21130 *g* for 5 min, and the absorbance (Abs) of each supernatant was measured at 540 nm using a spectrophotometer (Thermo Scientific Varioskan Flash Spectral Scanning Multimode Reader). The absorbance measured at 540 nm is directly proportional to the concentration of free hemoglobin in water.^33^ In some experiments, an erythrocyte pellet obtained from a 250 μL aliquot of blood was lysed in 400 μL of distilled water and used as a positive control. The untreated sample served as a negative control. A 10-fold dilution was performed on all samples and controls to ensure absorbance values were within a reliable range. The percentage hemolysis of a sample was calculated using eq 10. Data are presented as the mean ± standard deviation (SD) of triplicate samples from two independent donors with consistent results.

$$\mathrm{Hemolytic}\;\mathrm{index}\;(\%)=\left(\frac{\mathrm{Abs}\;\mathrm{sample}-\mathrm{Abs}\;\mathrm{negative}\;\mathrm{control}}{\mathrm{Abs}\;\mathrm{positive}\;\mathrm{control}-\mathrm{Abs}\;\mathrm{negative}\;\mathrm{control}}\right)\times 100$$ 10

### 2.12 Statistical Analysis

The results are presented as the mean ± SD from 3 to 4 replicates, with consistent results from 2 to 3 separate experiments. Statistical differences were analyzed using one-way ANOVA followed by Bonferroni’s post hoc test in SPSS software (SPSS Inc., Chicago, IL). A *p*-value ≤ 0.05 was considered statistically significant.

## 3 Results and Discussion

MRONJ is a severe adverse effect observed in patients undergoing prolonged treatment with high-dose antiresorptive medications like zoledronic acid (ZA). Due to the absence of clear clinical practice guidelines for MRONJ management, standard supportive care remains the primary approach. Our previous study demonstrated that surface functionalization of MCM-41 with 3-aminopropyltriethoxysilane (APTES) enhanced the ability of the resulting amine-modified MCM-41 (N-MCM-41) to adsorb and sustainably release poorly water-soluble geranylgeraniol (GGOH),^8^ capable of reversing ZA cytotoxicity at cellular and molecular levels.^7–9^ In this study, we report the development of dual-functional amine-modified Al-MCM-41 nanoparticles (N–Al-MCM-41) as a potential nanobiomaterial for preventing MRONJ associated with ZA. Functionalization with APTES enabled the nanoparticles to be simultaneously loaded with or releasing GGOH while adsorbing ZA. The physicochemical properties, GGOH-loading/releasing abilities, and ZA adsorption efficiency of both Al-MCM-41 and N–Al-MCM-41 were comparatively evaluated.

### 3.1 Characterization of Amine-Free and Amine-Containing Aluminum-Containing Mesoporous Silica Nanoparticles

After amine-functionalization using APTES, both starting (Al-MCM-41, AM) and resulting (N–Al-MCM-41, NAM) nanomaterials were comparatively characterized using XRD analysis, BET surface analysis of textural properties, and DLS measurement of particle size distribution and average sizes. The powder XRD patterns of the synthesized AM and NAM nanomaterials are presented in Figure S1 (in the Supporting Information (SI)). AM exhibited three characteristic diffraction peaks at 2θ values of 2.5°, 4.5° and 5.1°, corresponding to (100), (110) and (200) planes, confirming the presence of a well-ordered *p6* mm hexagonal structure of MCM-41.^25^ This suggested that after the incorporation of aluminum, the ordered mesoporous structure was still preserved. Upon APTES grafting, the main characteristic (100) peak at 2θ value of 2.5° remained observable with a decreased intensity, whereas the (110) and (200) peaks scarcely detected. This suggested that NAM still maintained the ordered hexagonal structure with a reduction in long-range order due to the incorporation of aminopropyl-functionalized groups within the mesoporous channels, which was consistent with the previous report.^34^ The results of BET analysis revealed significant decreases in both specific surface area from 752.80 m^2^/g to 115.16 m^2^/g and pore volume from 0.73 cm^3^/g to 0.16 cm^3^/g after Al-MCM-41 was amine-functionalized into N–Al-MCM-41. This was straightforwardly attributed to the bonding of APTES groups onto the Al-MCM-41 surface, including the pore surface (Table S1). Additionally, the pore size of the N–Al-MCM-41 nanomaterials appeared somewhat larger. The DLS results showed noticeable reductions in the median particle size (P50) from 154.89 ± 1.77 μm to 66.18 ± 1.26 μm and the largest particles (P90) from 410.22 ± 2.30 μm to 172.01 ± 0.92 μm after Al-MCM-41 was amine-functionalized (Table S2), indicating minimized aggregation of the N–Al-MCM-41 nanoparticles owing to electrostatic repulsion from surface-bound amine groups.

The surface morphology and elemental composition of the N–Al-MCM-41 nanoparticles were further examined using a scanning electron microscope equipped with energy dispersive X-ray analysis (SEM-EDX). Figure 1a displays an SEM image of N–Al-MCM-41, accompanied by its EDX elemental mapping images of carbon (C), nitrogen (N), oxygen (O), aluminum (Al), and silicon (Si); varied sizes of aggregates of nanoparticles were distinctly observed. Figure 1b discloses the determined elemental composition, the calculated atomic percentages of N, Al and Si, and the computed atomic ratio of N to Al of the nanomaterial, confirming the successful synthesis of Al-MCM-41 and amine functionalization of Al-MCM-41 using APTES. According to the measured percentages of N, Al and Si, 8% of degree substitution of Al in Al-MCM-41 was achieved.

**Figure 1:**
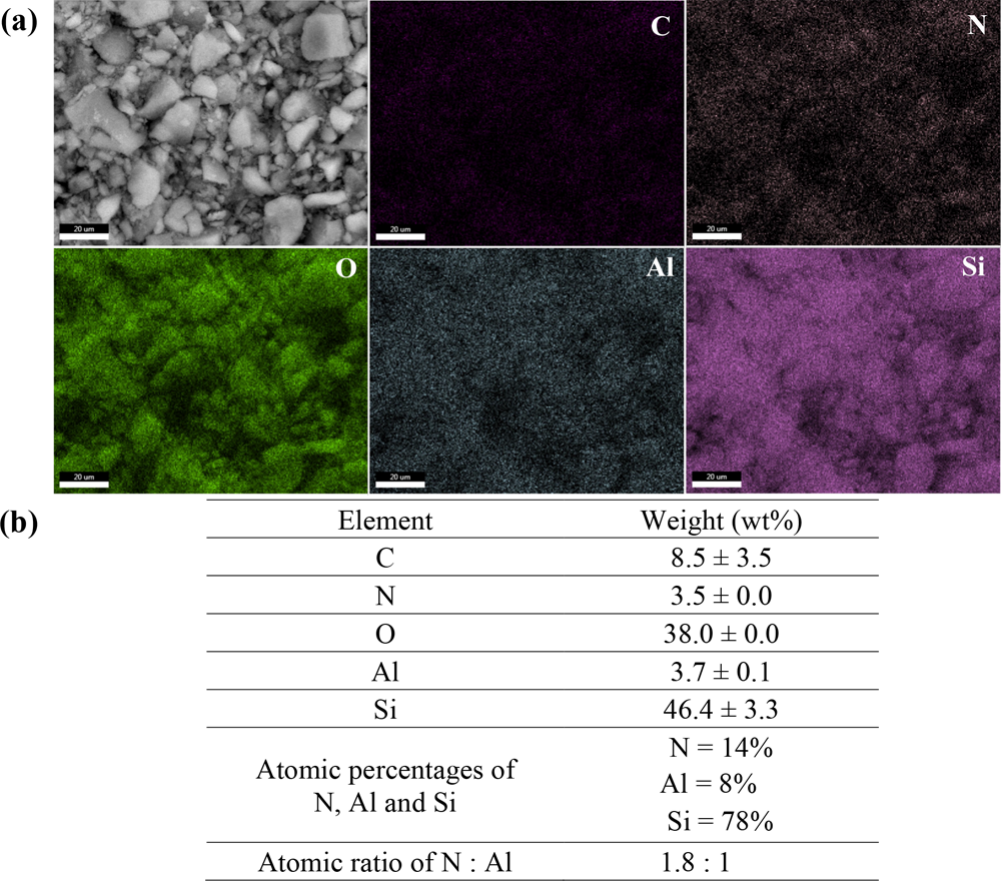
(a) SEM image and corresponding EDX elemental mappings of the N–Al-MCM-41 surface and (b) elemental composition of the nanomaterial, analyzed by SEM-EDX (*n* = 3). Scale bar = 20 μm.

### 3.2 Quantification of GGOH Loaded in Amine-Free and Amine-Containing Aluminum-Containing Mesoporous Silica Nanoparticles

In this study, three different GGOH-loaded nanoparticles, coded as 20G-AM, 0.5G-NAM, and 20G-NAM, were separately prepared by impregnating 20 mg of each dried nanomaterial in 1 mL of a 0.5- or 20-mM GGOH/EtOH solution at room temperature for 2 days in a shaking incubator at 130 rpm. To investigate the GGOH loading efficiency, all dried GGOH-loaded nanomaterials, along with their GGOH-free starting nanomaterials, were subsequently subjected to TGA analysis to collect their TGA thermograms over a temperature range of 30 to 800 °C. As presented in Figure S2, the overlaid TGA curves illustrate the changes in mass and phase transitions of the individual nanomaterials being combusted under sequential controlled atmospheres of N_2_ and O_2_. The initial mass loss of each test sample between 30 and 150 °C was primarily attributed to physically absorbed moisture. Figure 2 reveals the corresponding derivative thermogravimetric (DTG) thermograms of the combusted specimens in the temperature range of 150–800 °C, while Table 1 demonstrates the weight losses (%w/w) of the individual key components in each test specimen. A tiny mass reduction (1.62%) of GGOH-free aluminum-doped mesoporous silica nanoparticles (AM) was observed at the elevated temperatures ranging from 550 to 800 °C which was associated with the loss of water formed through the condensation reaction between free silanol groups (−Si–OH) on the surface of the nanoparticles,^35^ leaving a substantial mass (98.38%) of silica residue after the analysis. Apart from the small combustion with a weight loss of 1.98% at 550–800 °C, an additional mass loss (10.86%) of GGOH-free amine-functionalized aluminum-doped mesoporous silica nanoparticles (NAM) was markedly detected at 250–550 °C which was attributed to the decomposition of the organic aminopropyl groups grafted onto NAM after the amine-functionalization of AM with APTES;^36^ the residual mass of NAM found at 800 °C was then reduced to 87.16%. After GGOH was integrated into both AM and NAM, the TGA thermograms of all GGOH-loaded nanocarriers, particularly that of 20G-NAM, markedly disclosed a thermal degradation of GGOH principally at temperatures around 150–250 °C with a miniscule combustion area overlapping with that of the organic aminopropyl groups. The presence of aminopropyl groups on the surface of NAM turned this nanomaterial to be more hydrophobic, strikingly boosting the adsorption of the poorly water-soluble GGOH onto the NAM surface;^37,38^ an approximately 4-fold increase in the amount of GGOH actually loaded in 20G-NAM (12.48%) was found, compared to that detected in 20G-AM (3.04%). Even when a substantially lower concentration of GGOH initially employed in the GGOH-loading process of NAM, i.e., 0.5 mM GGOH/EtOH solution, the weight percentage of GGOH essentially determined in 0.5G-NAM (1.90%) was slightly less than that of 20G-AM. The residues of the GGOH-loaded NAM particles found at 800 °C were successively subsided, compared to that of GGOH-free NAM, owing to the presence of the increasing amount of GGOH loaded in the nanomaterial.

**Figure 2:**
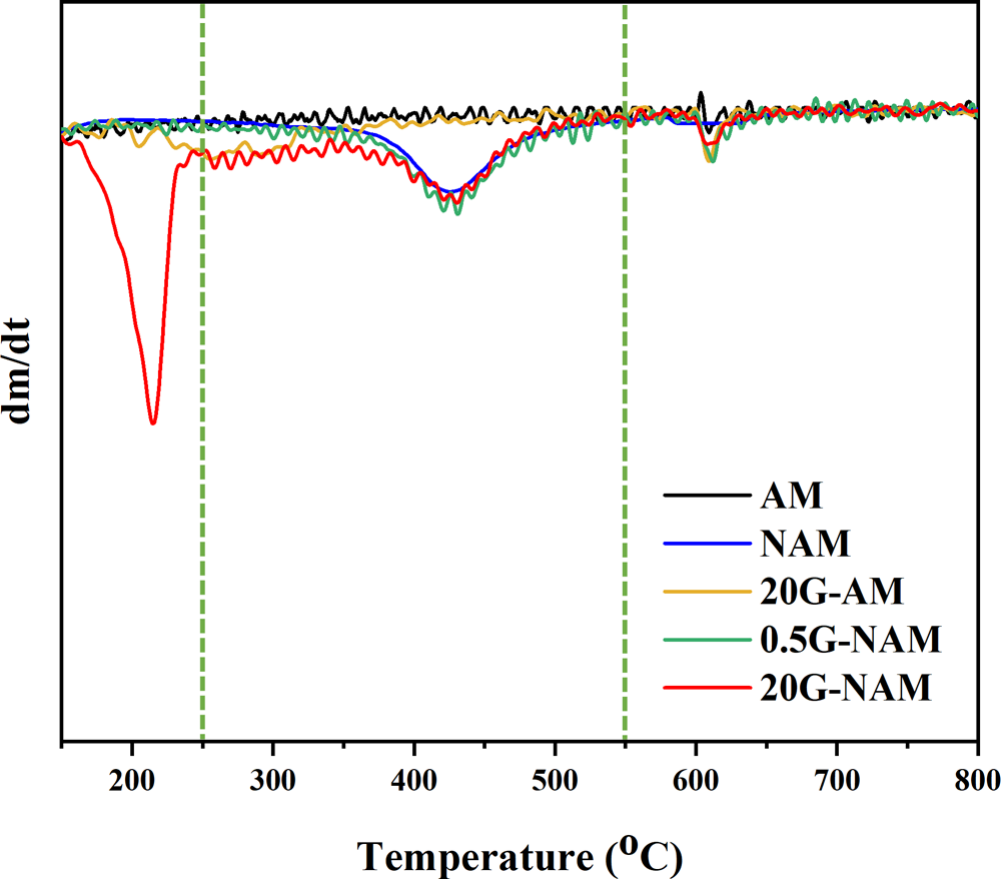
Overlaid DTG curves of the GGOH-free and GGOH-loaded nanomaterials.

**Table 1:** TGA Results of the GGOH-Free and GGOH-Loaded Nanomaterials (*n* = 1)

	weight loss at transition (%)			
sample	150–250 °C	250–550 °C	550–800 °C	residual weight (%) at 800 °C
AM	ND	ND	1.62	98.38
NAM	ND	10.86	1.98	87.16
20G-AM	3.04	6.73	1.74	88.49
0.5G-NAM	1.90	11.40	2.28	84.42
20G-NAM	12.48	13.95	1.97	71.60

### 3.3 In Vitro GGOH Release Profiles

The profile of GGOH liberated from each nanomaterial was directly determined by HPLC after the supernatants of each GGOH-loaded specimen (20 mg, *n* = 2 except for 20G-AM) immersed in 1.2 mL of PBS mixed with 10% (v/v) FBS at 37 °C were daily collected for 10 days. The amounts of GGOH in the individual test samples were promptly calculated by correlating the peak areas in their HPLC chromatograms to the GGOH standard curve and then reported in the following terms: the percentage of GGOH totally released per weight of a GGOH-loaded carrier (*R*_G,HPLC_), the cumulative GGOH release per weight of a GGOH-loaded carrier (*C*_G,C_), the cumulative GGOH release per total amount of GGOH actually loaded (*C*_G,G_), and the percentage of GGOH remaining per weight of a GGOH-loaded carrier (*L*_G,HPLC_), computed using the eqs 1–4 listed in Section 2.7.

As shown in Table 2, both AM and NAM nanomaterials could prolongedly free GGOH up to 10 days with relatively much smaller quantities of GGOH daily liberated from both 20G-AM and 0.5G-NAM, compared to that of 20G-NAM. This agreed with the TGA results that revealed the greatest amount of GGOH in 20G-NAM. Also, this confirmed that the amine-functionalized Al-MCM-41 nanoparticles explicitly served as a good carrier for GGOH loading and controlled-releasing. Moreover, there were noticeable burst releases of GGOH from the GGOH-loaded NAM nanomaterials on the first 4 and 3 days of releases, accounting for about 56% (Day 1–Day 4) and 58% (Day 1–Day 3) of the total amount of GGOH freed from 0.5G-NAM and 20G-NAM, respectively, followed by a nearly plateau stage of GGOH liberations up to 10 days. The burst release was primarily caused by the diffusion of GGOH vastly accumulated at the outer surfaces of nanoparticles, while the subsequent sustained release was plausibly governed by the GGOH diffusion from the internal pores of nanoparticles where there existed a GGOH-carrier interaction. During this 10-day assessment, the total amount of GGOH freed from 20G-NAM was approximately 26 times higher than that released from 0.5G-NAM although the initial loading concentration of GGOH used in the preparation of 20G-NAM was 40 times as much as that exploited for 0.5G-NAM. A prolonged release study would positively reveal a greater gap in the total quantities of GGOH freed from these two GGOH-loaded nanocarriers. Unexpectedly, the release profile of GGOH from 20G-AM appeared somewhat different from that of the GGOH-loaded NAM nanomaterials; a nearly constant concentration of GGOH was daily freed from this amine-free nanomaterial. No GGOH burst release was observed in this material. Nevertheless, the ratio of the total amounts of GGOH liberated from 20G-AM (∼95 μg) and 0.5G-NAM (∼66 μg) within 10 days was fairly close to the ratio of the amounts of GGOH found in these two nanomaterials, determined by TGA.

**Table 2:** Release Profiles and Total Released Amounts of GGOH from the Individual GGOH-Loaded Nanocarriers Quantified by HPLC Analysis (*n* = 2)

	released GGOH concentration (μΜ)		
day	20G-AM^a^	0.5G-NAM	20G-NAM
1	19.8	30.9 ± 1.5	1134.1 ± 194.0
2	29.7	28.5 ± 0.6	955.5 ± 66.4
3	25.1	26.7 ± 1.4	744.1 ± 120.3
4	18.8	20.5 ± 0.8	272.3 ± 56.5
5	24.2	12.8 ± 0.8	232.6 ± 12.8
6	26.4	19.0 ± 0.6	306.1 ± 57.7
7	29.3	15.9 ± 0.2	319.5 ± 1.5
8	32.3	14.6 ± 1.0	338.3 ± 17.4
9	34.1	10.7 ± 0.7	321.8 ± 25.4
10	32.6	9.4 ± 1.4	296.6 ± 7.8
Total amount of GGOH released (μg)	94.9	65.9 ± 0.9	1715.3 ± 23.0

a *n* = 1.

After being immersed in the PBS/FBS mixed medium for 10 days, all dried GGOH-loaded specimens, coded as 20G-AM-Day10, 0.5G-NAM-Day10, and 20G-NAM-Day10, respectively, were separately subjected to TGA analysis to determine the weight percentage of GGOH left in each nanomaterial (*L*_G,TGA_), and the weight percentage of GGOH totally released from each carrier (*R*_G,TGA_) was subsequently calculated by subtracting *L*_G,TGA_ from *T*_G,TGA_. Table 3 presents the weight percentages of GGOH actually loaded, released (within 10 days), and remaining per weight of GGOH-loaded nanoparticles and per total amount of GGOH actually loaded, determined by both TGA and HPLC analyses. Since the combustion of GGOH left in both 20G-AM-Day10 and 0.5G-NAM-Day10 was scarcely detected in their TGA thermograms (data not shown), the *R*_G,TGA_ values of these materials could not be estimated. On the other hand, about 6.33% GGOH was decomposed in the TGA curve of 20G-NAM-Day10 (data not shown); the *R*_G,TGA_ value of 20G-NAM was thus approximated to be 6.15% (its *T*_G,TGA_ = 12.48% (Table 1)). This TGA-determined value was somewhat close to that (8.58%) analyzed by HPLC. Noteworthy, after being soaked in the PBS/FBS mixed medium for 10 days, about 50% (determined by TGA) or 69% (measured by both HPLC and TGA) of GGOH actually loaded into the 20G-NAM nanoparticles had been liberated into the medium with a burst release (58%) of GGOH during the first 3 days of measurement. According to both release profile and quantity, this excessively GGOH-loaded nanomaterial was presumably expected to prolongedly free GGOH up to at least 20 days with some remaining GGOH entrapped in the nanocarrier. A GGOH release study with an extended period is needed in the future to justify this anticipation.

**Table 3:** Weight Percentages of GGOH Actually Loaded, Released, and Left Per Weight of GGOH-Loaded Nanocarrier and Per Total Amount of GGOH Actually Loaded, Determined by Both TGA and HPLC Analyses

	TGA			HPLC	
sample	*T*_G,TGA_ (%)	*L*_G_,_TGA_ (%)	*R*_G,TGA_ (%)	*R*_G,HPLC_ (%)	*L*_G,HPLC_ (%)
20G-AM	3.04^a^	ND	ND	0.47^a^	2.57^a^
	100^b^			15.61^b^	84.39^b^
0.5G-NAM	1.90^a^	ND	ND	0.33^a^	1.57^a^
	100^b^			17.37^b^	82.63^b^
20G-NAM	12.48^a^	6.33^a^	6.15^a^	8.58^a^	3.90^a^
	100^b^	50.72^b^	49.28^b^	68.75^b^	31.25^b^

ND = not detected. Superscript “a” indicates the total amount of GGOH reported per weight of a GGOH-loaded carrier (see the equations in Section 2.7), while superscript “b” indicates the total amount of GGOH reported per total amount of GGOH actually loaded, determined by TGA.

Figure 3 demonstrates the cumulative GGOH release (%) per weight of a GGOH-loaded carrier (*C*_G,C_) (blue line) and the cumulative GGOH release (%) per total amount of GGOH actually loaded (*C*_G,G_) (red line) as a function of release time. As demonstrated in Table 1, the total amounts of GGOH liberated from the nanocarriers within 10 days were relatively much lower than the quantity of NAM (20 mg) initially exploited in the GGOH-loading process; the *C*_G,C_ values of these GGOH-loaded nanomaterials were, therefore, rather small, particularly those of 20G-AM and 0.5G-NAM where their *C*_G,C_ values on Day 10 reached only 0.47% and 0.33%, respectively, (Table 3 and Figure 3) owing to the surface hydrophilicity of AM nanoparticles unfavorable for GGOH loading and a loading of GGOH at a relatively low concentration in case of 0.5G-NAM. Apparently, the plots of both *C*_G,G_ and *C*_G,C_ versus releasing time of 20G-NAM exhibited two distinctly different slopes with a steeper slope during the first 3 days of release, indicating a burst liberation of GGOH. Afterward, a relatively slower and rather steady release of GGOH from this material was perceived from Day 4 onward. On the contrary, those of both 20G-AM and 0.5G-NAM looked more linear-like, particularly those of 20G-AM, suggesting a nearly negligible burst release. This was partially attributed to the relatively far lower quantities of GGOH daily released from the carriers.

**Figure 3:**
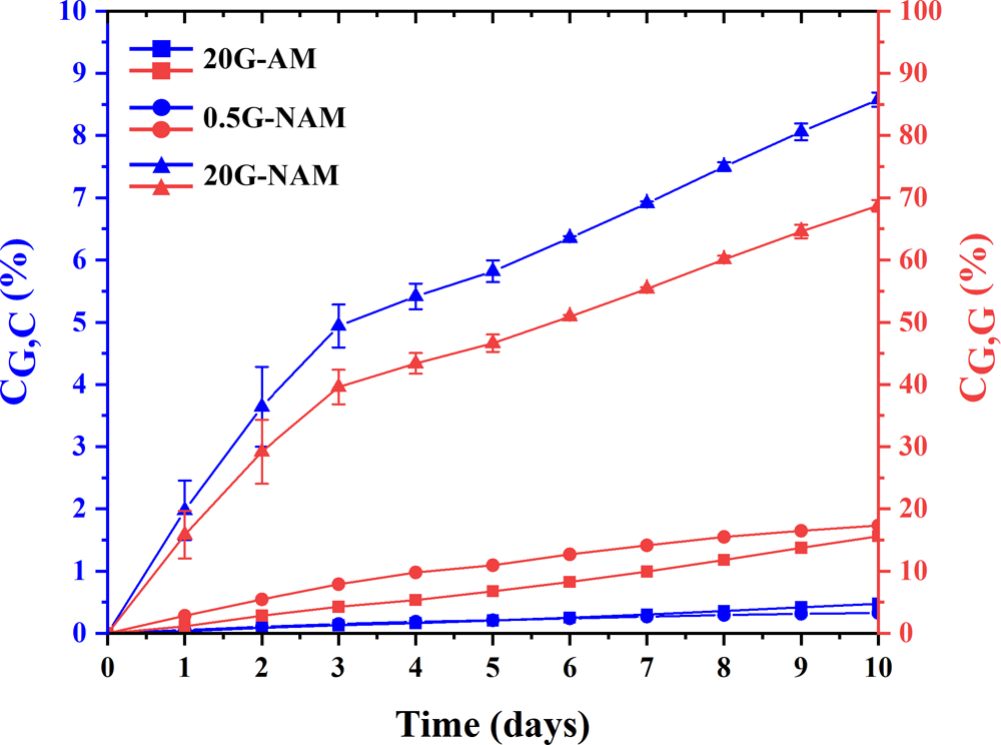
Cumulative GGOH release (%) per weight of a GGOH-loaded carrier (*C*_G,C_) and the cumulative GGOH release (%) per total amount of GGOH actually loaded (*C*_G,G_) as a function of releasing time of each GGOH-loaded nanocarrier.

### 3.4 In Vitro GGOH Release Kinetics and Mechanisms

The study of drug release kinetics is of importance in drug delivery systems as they govern drug release mechanisms and rates. The release kinetics are typically examined via experimental validation employing mathematical modeling. In the present study, four mathematical models, namely First-order, Higuchi, Korsmeyer-Peppas, and Hixson-Crowell kinetic models (see their equations in Section 2.8), were individually evaluated to describe the release kinetics of GGOH freed from the amine-functionalized and nonfunctionalized aluminum-doped mesoporous silica nanoparticles using the corresponding GGOH release profiles.

Figure 4 presents the linear regression plots of GGOH release as a function of release time (day) of each GGOH-carrying nanocarrier, individually assessed by all four mathematical models. Although the whole-lifetime GGOH release profiles of both 0.5G-NAM and 20G-NAM displayed a biphasic release pattern comprising (fairly) burst and (rather) steady releases, the entire release data could be impeccably fitted by using a single equation, generating linear plots with high regression coefficients (*R* ^2^). Among all mathematical models applied, the Higuchi model generated the best fitting curves of the GGOH release data of both 0.5G-NAM and 20G-NAM with the greatest regression coefficients of 0.9988 and 0.9868, respectively (Figure 4b). This suggested that the release mechanism of GGOH from the NAM nanoparticles was primarily a Fickian diffusion-controlled release;^39^ GGOH trapped in the nanopores prolongedly diffused into the releasing medium. In addition, the release mechanism of GGOH from this type of mesoporous silica nanoparticles consequently appeared independent of the amount of GGOH loaded. However, the GGOH release rates of these two materials demonstrated a concentration-dependent release behavior, which should not be observed when both exhibited the same release mechanism, i.e., Higuchi. As revealed in Figure 4a, the regression coefficient (0.9856) of linear plot of 20G-NAM release data fitted by the First-order model was very close to that (0.9868) fitted by the Higuchi model. Probably, the release mechanism of GGOH from 20G-NAM could be codetermined by both Higuchi and First-order where the rate of drug release is dependent on the amount of drug loaded in a carrier. It was also noted that the linear regression plot created by the Korsmeyer-Peppas model was most perfectly fitted to the GGOH release profile of 20G-AM with a regression coefficient of 0.9960. In Korsmeyer-Peppas model, the drug release mechanism for a spherical-shaped carrier can be classified into four different release phenomena based on a calculated release exponent value (*n*) which is the slope of a plot; *n* = 0.43 for Fickian diffusion, 0.43 < *n* < 0.85 for non-Fickian diffusion or anomalous transport, *n* = 0.85 for Case-II transport, and *n* > 0.85 for Super Case-II transport.^40^ As shown in Figure 4c, the calculated *n* value of GGOH released from 20G-AM was 1.10, belonging to a Super Case-II transport process where the drug transport is controlled primarily by swelling of a drug-carrier^41^ which was unlikely applicable to the mesoporous silica nanoparticles. Hence, the release mechanism of GGOH from 20G-AM best fitted by the Super Case-II transport model could be plausibly associated with the less restricted chemical structure of the amine-free nanocarrier, enabling the diffusion-controlled process in the absence of the interaction between GGOH and the aminopropyl groups, allowing the steady delivery of GGOH. Nevertheless, this result was gathered from an *n* = 1 experiment.

**Figure 4:**
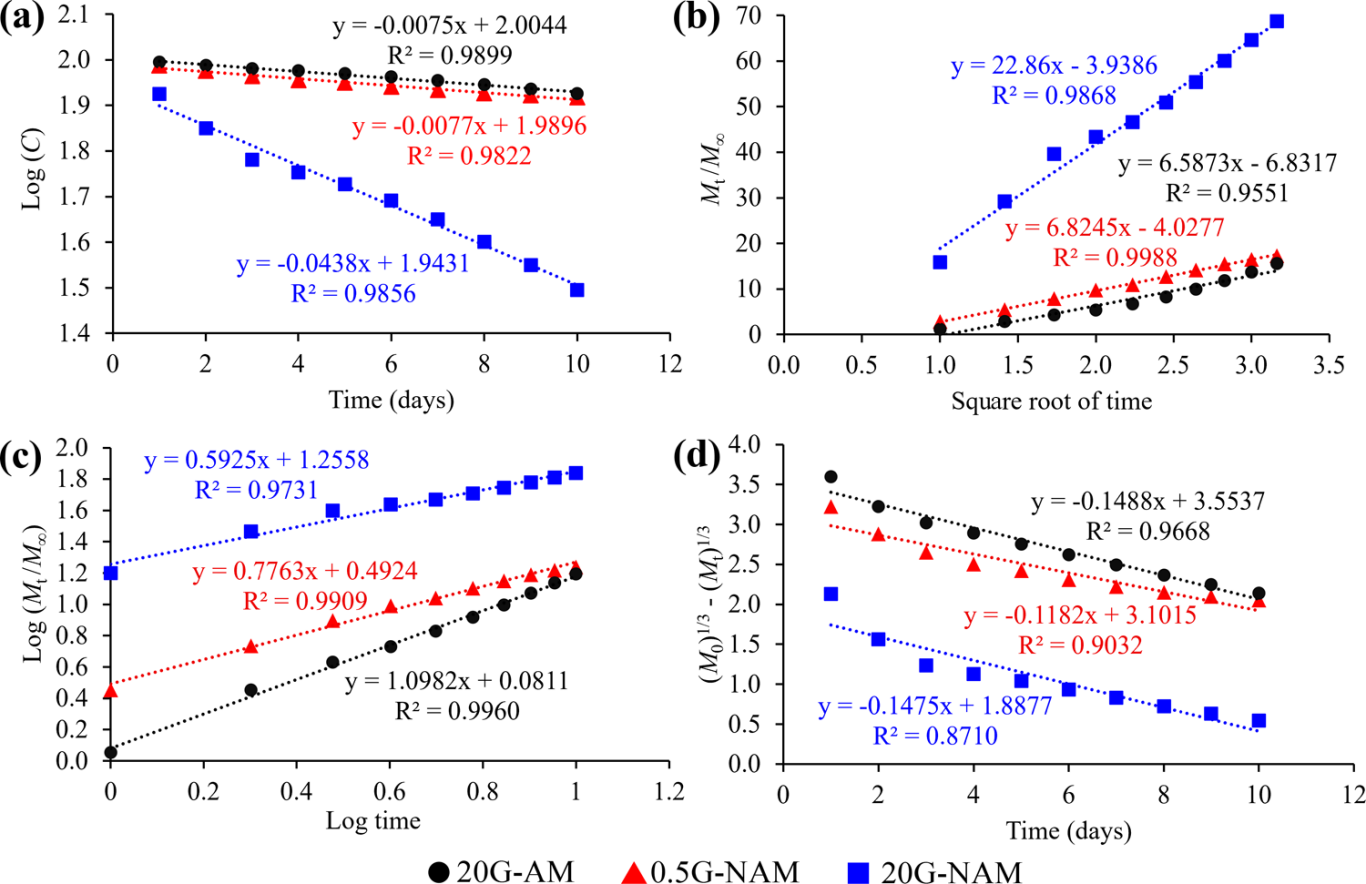
Linear regression plots constructed from the GGOH release profile of each GGOH-loaded nanocarrier using (a) first-order, (b) Higuchi, (c) Korsmeyer–Peppas, and (d) Hixson–Crowell models.

### 3.5 ZA Adsorption Efficiency

ZA effectively suppresses osteoclast-mediated bone resorption by inhibiting the mevalonate pathway, reducing bone turnover, and stabilizing bone structure. However, excessive suppression may hinder microdamage repair, increasing the risk of atypical femoral fractures and bisphosphonate-related osteonecrosis of the jaw (BRONJ), particularly in cancer patients or following dental interventions. Since excess ZA may worsen BRONJ risk,^9,42^ using drug carriers to adsorb surplus ZA is expected to minimize off-target effects, such as renal toxicity and BRONJ, while improving therapeutic outcomes.

This study comparatively assessed the ZA adsorption efficiency of the GGOH-free and GOOH-loaded nanocarriers after 1, 2, and 3 days of immersion in 100 μM ZA in DI water. As demonstrated in Table 4, among all test nanomaterials, AM exhibited the highest ZA adsorption ability, reaching nearly 90 and 95% after 1 and 2 days of immersion, respectively, which was absolutely attributed to the strong binding affinity between the phosphonate groups in ZA and the substituted aluminum ions in the nanocarrier.^17,18^ At Day 3, its ZA adsorption value remained unchanged, indicating the maximum ZA adsorption saturation in the AM nanocarrier. After amine functionalization, the considerably lower ZA adsorption efficiency of NAM was observed with an initial adsorption of 14% on Day 1, followed by gradually increasing to 31% on Day 3. The presence of aminopropyl groups in NAM explicitly hindered the binding between ZA and Al in NAM. After loading GGOH into NAM at different GGOH concentrations, the ZA adsorption efficiency of the 0.5G-NAM and 20G-NAM nanocarriers almost scarcely altered, compared to that of the corresponding GGOH-free nanomaterial, denoting that the presence of GGOH in this type of mesoporous silica nanomaterial had a negligible effect on the ZA adsorption efficiency.

**Table 4:** ZA Adsorption Efficiency of the GGOH-Free and GOOH-Loaded Nanomaterials after 1, 2, and 3 Days of Immersion (*n* = 2)

	ZA adsorption (%)			
day	AM	NAM	0.5G-NAM	20G-NAM
1	87.40 ± 4.70	13.72 ± 6.21	15.58 ± 0.39	11.93 ± 0.04
2	94.40 ± 0.30	24.36 ± 1.59	19.50 ± 0.89	31.41 ± 0.62
3	95.05 ± 2.15	30.90 ± 1.60	25.46 ± 2.12	24.30 ± 2.40

### 3.6 In Vitro Cytocompatibility and Cytoprotective Activity against ZA

It was clearly evident that the amine-functionalized Al-MCM-41 nanoparticles served as a good carrier for GGOH loading and controlled-releasing, and 20G-NAM could carry and daily release GGOH at far larger amounts than 0.5G-NAM. As a result, the biological property of this GGOH-loaded nanomaterial was subsequently assessed. Figure 5 shows that in the untreated control group, cells exhibited strong CFSE staining (green cytoplasm), indicating healthy, live cells with minimal PI-positive dead cells. Cells treated with 20G-NAM at all concentrations tested showed minimal impact on the number of viable cells with bright CFSE staining and limited PI staining. Notably, in cultures treated with 20G-NAM at 200 and 300 μg/mL, cells formed multicellular clusters. Additionally, two subpopulations of unhealthy but viable cells (CFSE-dull/negative and PI-negative) and dead cells (PI-positive) were observed in the 300 μg/mL nanomaterials treatment group. Treatment with ZA alone resulted in a significant decrease in the number of viable cells with less than half of the cells remaining adherent. This was accompanied by a marked decrease in CFSE staining and a significant increase in PI staining, indicating increased cell death compared with the untreated control. Importantly, cotreatment with ZA and 20G-NAM resulted in significant preservation of viable cells compared with ZA treatment alone. All three concentrations of 20G-NAM appeared to possess similar high efficiency in mitigating ZA-induced cell death.

**Figure 5:**
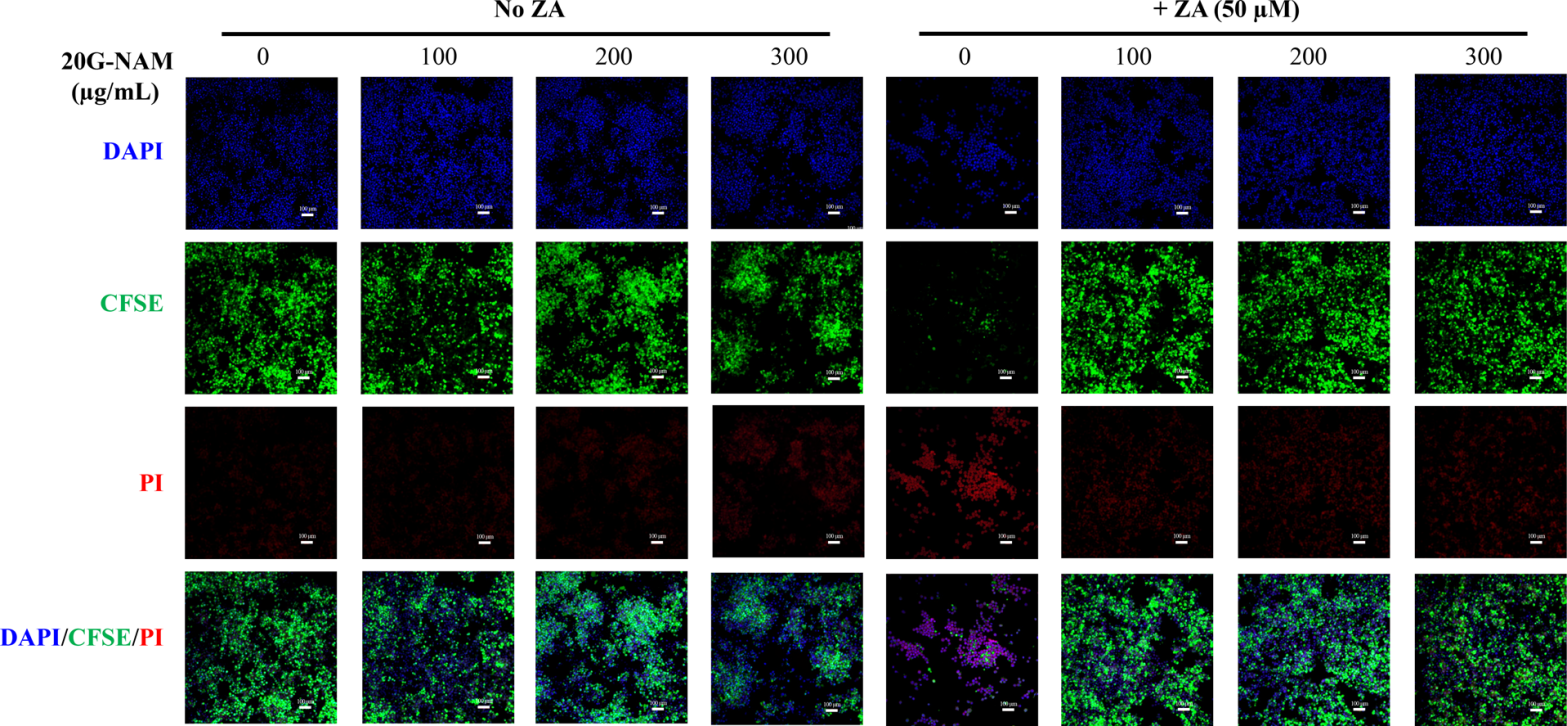
Representative confocal fluorescence images of live/dead cell staining of RAW cells to assess the cytocompatibility of 20G-NAM and its cytoprotective effect against ZA-induced cytotoxicity. Cells were treated with various concentrations of 20G-NAM (100–300 μg/mL) without and with ZA (50 μM) for 72 h. Cells were then stained with DAPI (blue, nuclei), CFSE (green, live cells), and PI (red, dead cells). Scale bar: 100 μm.

In Figure 6, the resazurin assay revealed that treatment with 20G-NAM at all three concentrations tested did not affect cellular metabolic activity, suggesting the cytocompatibility of the material with RAW cells at concentrations of 100–300 μg/mL. ZA alone significantly and markedly decreased metabolic activity, while cotreatment with 20G-NAM attenuated this effect. Interestingly, 20G-NAM at 100–300 μg/mL reversed the reduced metabolic activity of the ZA-treated cells from approximately 30% to a level similar to that of the untreated control cells. The findings indicated that within the concentration range used, the 20G-NAM nanoparticles were cytocompatible without affecting metabolic activity and demonstrated a complete cytoprotective effect against ZA-induced impaired metabolic activity in RAW cells.

**Figure 6:**
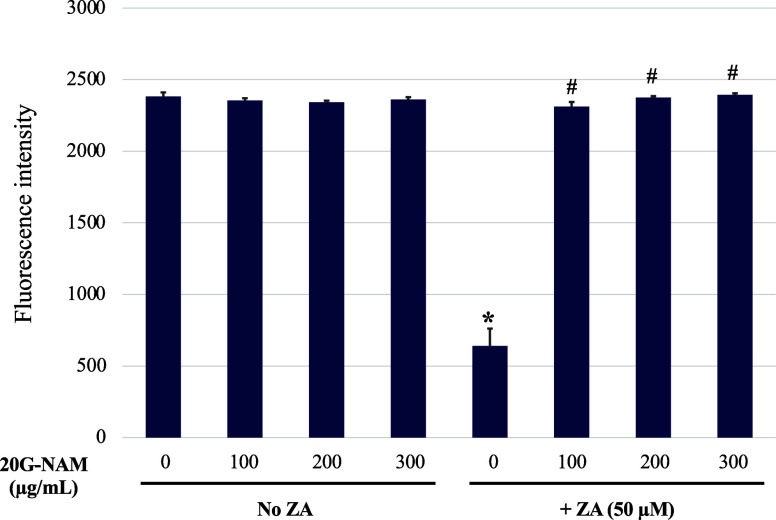
Effect of 20G-NAM on metabolic activity and ZA-reduced metabolic activity in RAW cells. RAW cells were treated with various concentrations of 20G-NAM (100–300 μg/mL) in the absence and presence of ZA (50 μM) for 72 h. Metabolic activity was assessed using the resazurin assay. Data are expressed as mean ± SD (*n* = 3) *p < 0.05 vs the untreated control group. #*p* < 0.05 vs the ZA-treated group (in the absence of 20G-NAM).

Given that 20G-NAM at 100–200 μg/mL was found to be noncytotoxic and completely reversed ZA-induced cytotoxicity to RAW cells, the effect of the nanoparticles on apoptosis prevention was subsequently determined at these concentrations. In Figure 7, flow cytometric analysis of annexin V/PI staining revealed significant differences in cell viability and apoptotic distribution among the treatment groups after 24 h. Control cells exhibited high viability with minimal early and late apoptosis. ZA treatment significantly reduced viability to approximately 82%, while increasing early apoptosis to 12% and late apoptosis to 5%. ZA-induced necrosis was detected at only 1%, confirming that ZA-induced cytotoxicity occurred predominantly via apoptosis, as previously reported.^7^ Cotreatment with 20G-NAM nanoparticles markedly improved cell apoptosis. At 100 μg/mL, viability increased to 95% with early apoptosis and late apoptosis reduced to 2.5% and 1.8%, respectively. A stronger protective effect was observed at 200 μg/mL, where viability increased to 98%, a level similar to that of the untreated control cells, and apoptosis fractions were nearly identical to those of the control (early apoptosis, 1.2%; late apoptosis, 0.9%). The findings indicated that 20G-NAM significantly rescued ZA-induced apoptosis in RAW cells.

**Figure 7:**
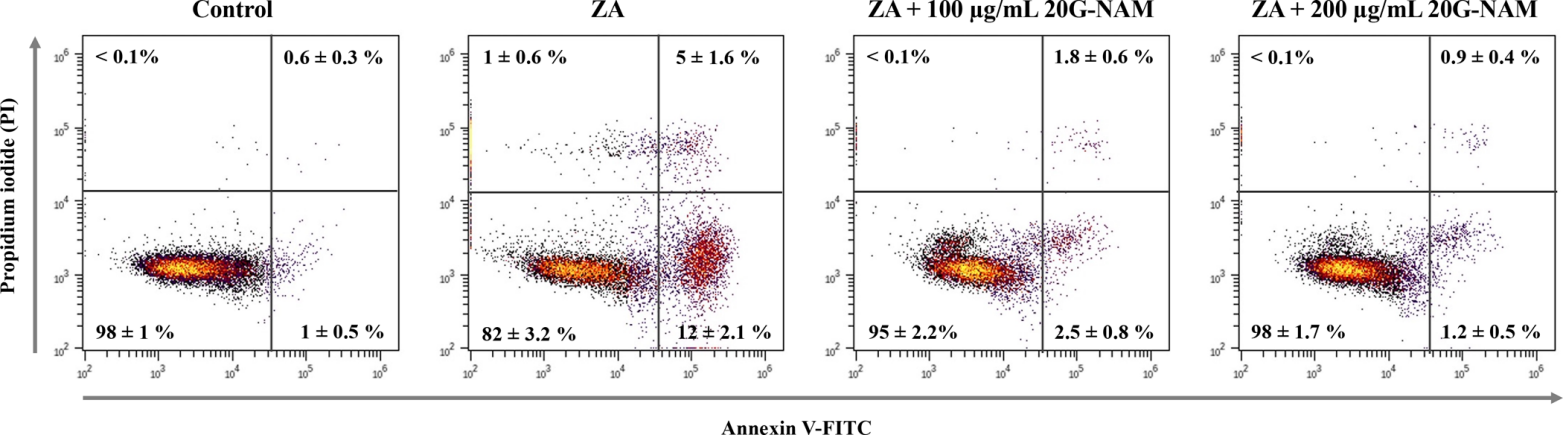
Representative flow cytometric dot plots of annexin V-FITC and propidium iodide (PI) of RAW cells to assess the apoptosis reversal activity of 20G-NAM against ZA. Cells were treated for 24 h with vehicle control, ZA (50 μM), or ZA in combination with 20G-NAM (100 or 200 μg/mL). Cells were stained with annexin V-FITC and PI to distinguish viable cells (annexin V^–^/PI^–^), early apoptotic cells (annexin V^+^/PI^–^), late apoptotic cells (annexin V^+^/PI^+^), and necrotic cells (annexin V^–^/PI^+^). Representative dot plots are shown with mean percentage values of cells ± SD in each quadrant (*n* = 3). When comparing the percentage of cells in the same quadrant, no significant differences were found only among the control, ZA + 100 μg/mL 20G-NAM, and ZA + 200 μg/mL 20G-NAM groups.

RAW cells are monocyte/macrophage-liked cells that are highly fusogenic cells capable of forming syncytial multinucleated giant cells, such as those observed in response to foreign bodies. In RAW cells treated with 20G-NAM at 200–300 μg/mL in the cytocompatibility experiment, the multicellular clusters may be attributed to their role in phagocytosing large 20G-NAM aggregates that individual RAW cells could not engulf,^43^ as seen in our preliminary toxicity test demonstrating that RAW cells treated with bare (GGOH-free) Al-MCM-41 nanoparticles at a nontoxic concentration formed multicellular clusters of singlet RAW cells with multinucleated giant cells nearby (Figure S3). Despite ultrasonic dispersion prior to cell treatment, nanoparticle aggregation persisted in the culture containing high concentrations of 20G-NAM (Figure S4). The potential impact of unavoidable aggregated nanoparticles on cellular viability, function, and cytoprotective effects against ZA remains uncertain, particularly when considering their intracellular fate in comparison to extracellular nanoparticles. Further research is necessary to elucidate the precise mechanisms by which 20G-NAM regulates multinucleated giant cell formation and to assess the significance of nanoparticle-induced multinucleated giant cells in preventing MRONJ. While the experimental design may limit a full exploration of the underlying mechanisms, the present study demonstrates a net positive effect of both intracellular and extracellular 20G-NAM nanoparticles on cell viability and cytoprotection against ZA in RAW cells.

Neither of the two assays, even under extended incubation times (72 h), indicated significant cytotoxicity and impaired metabolic activity associated with 20G-NAM, suggesting that the nanoparticles used in this study were nontoxic at the concentration range used herein (100–300 μg/mL). The 20G-NAM nanoparticles at this concentration range also fully mitigated ZA-induced cytotoxicity and suppression of metabolic activity in RAW cells. This cell line is a well-established precursor of activated macrophages and osteoclasts, both of which play a crucial role in the pathogenesis of MRONJ. An optimal concentration of 20G-NAM for preventing ZA-suppressed cell viability should also be nontoxic and allow these cells to exert their normal functions in the absence of ZA exposure. This concentration appeared to be <300 μg/mL. Live/dead cell staining indicated that 20G-NAM at 300 μg/mL caused some RAW cells to become unhealthy and even dead (Figure 5), despite not affecting cellular metabolic activity (Figure 6). It is imperative to recognize that metabolic activity, while indicative of cellular function, is not necessarily similar to cell viability.^44^ It is possible that while 20G-NAM at 300 μg/mL reduced the viable cell population, the remaining viable cells, including those forming multinucleated giant cells, have increased metabolic activity, resulting in unaffected total metabolic activity. Therefore, the concentration range below 300 μg/mL may be used to explore the role of 20G-NAM in preventing ZA-suppressed relevant functions of macrophages and osteoclasts. A previous study using primary human macrophages demonstrated that mesoporous silica nanoparticles at a dose of 1–200 μg/mL did not impair cell viability or MRONJ-related functions, including cytokine secretion and efferocytosis.^45^

Cotreatment with 20G-NAM rescued cell apoptosis in a dose-dependent manner. At 100 μg/mL, 20G-NAM reduced both early and late apoptosis by more than 70% compared with ZA alone. At 200 μg/mL, viability was restored to levels indistinguishable from those of untreated controls. The observation that early apoptosis remained consistently higher than late apoptosis across all treated groups reflected a protective mechanism of 20G-NAM that interrupted the progression of apoptosis at its initial stage. The absence of necrosis across all conditions indicates that ZA toxicity and its reversal by 20G-NAM operate primarily through apoptosis-related pathways without significant membrane disruption.^7^ This is consistent with clinical relevance, as ZA-associated osteonecrosis of the jaw (MRONJ) is believed to involve impaired osteoclast function and enhanced apoptosis rather than direct necrotic cell death.^1^

While 20G-NAM, as submicron-sized mesoporous silica particles, may be highly internalized by primary human macrophages,^45^ the extent to which this influences their ability to prevent ZA-induced cellular dysfunction remains to be fully elucidated. Preliminary determination of cellular uptake of 20G-NAM initially demonstrated that only a small proportion of cells (12%) could uptake 20G-NAM by an energy-dependent internalization pathway, but not by free diffusion (Figure S5). This suggested that extracellular 20G-NAM might make a major contribution to the reversal of ZA-induced cytotoxicity to RAW cells. A more detailed study is needed for a better understanding of the cellular uptake, intracellular fate, and exocytosis of the dual-action 20G-NAM nanoparticles. Such data directly impact the efficacy, safety, and ultimate clinical translatability of the nanoparticle system.

GGOH concentrations of 80 μM or higher exhibited cytotoxicity toward osteoclast precursors following a 7-day treatment period.^46^ In vitro studies have demonstrated a cytoprotective effect of GGOH against ZA within an optimal concentration range. However, both lower and higher concentrations of GGOH were associated with reduced cytoprotective effects and increased cell death, respectively.^8,11,46^ It has been proposed that the GGOH-to-ZA concentration ratio should ideally not exceed 100%.^11^ Notably, GGOH could only rescue cell viability and biological function up to a certain threshold.^46^ GGOH released from a hydrogel at 2.5–65 μM only partially mitigated the cytotoxicity induced by 5 μM ZA, restoring cell viability to approximately 70% of the untreated control level.^8^ In the present study, 20G-NAM nanoparticles demonstrated a complete (100%) reversal of ZA-induced cytotoxicity, highlighting the biological significance of 20G-NAM-mediated ZA adsorption. Under the tested condition used in the ZA adsorption experiment, the estimated maximum ZA adsorption capacity of 20G-NAM nanoparticles was approximately 13 μg ZA/100 μg nanoparticles. Although the aluminum-to-bisphosphonate molar ratio in the present adsorption interaction remains unknown, bisphosphonates have been shown to form complexes with aluminum with 1:1 or 1:2 aluminum-to-bisphosphonate molar ratio at pH above 4 or pH below 4, respectively.^17^ In addition, the estimated maximum ZA adsorption capacity of the corresponding AM nanoparticles without GGOH loading was approximately 3 folds higher than that of 20G-NAM. These data suggest that further optimization may help increase ZA adsorption efficiency while effectively maintaining the release profile of GGOH at an optimal concentration range discussed above. The high efficacy of 20G-NAM in fully mitigating ZA toxicity also suggests that both ZA adsorption via aluminum functionalization and the release of GGOH remain cooperatively functional and highly active under the cell culture conditions used (i.e., nutrient-rich medium, supplemented with inorganic salts and 10% serum at pH 7.2–7.4). Whether internalized 20G-NAM retains its efficient ZA adsorption and GGOH delivery capacities within the intracellular microenvironment, which differs significantly from the extracellular environment, remains a challenging area of investigation.

### 3.7 Erythrocyte Compatibility

Hemolytic activity is a critical safety concern for nanomaterials intended for biomedical applications that come into contact with red blood cells, the most abundant type of blood cell in humans. Mesoporous silica nanoparticle-erythrocyte interaction occurs in different ways, including nanoparticle adhesion to the surface of red blood cells without damaging the membrane or shape, local membrane deformation of red blood cells, and red blood cell hemolysis.^47^ The level of plasma membrane damage depends on the mesoporous silica nanoparticle concentration and the level of cellular adhesion of the nanoparticles, which generally occurs via the interaction between the surface silanols of mesoporous silica nanoparticles and the erythrocyte membrane.^47,48^ However, smaller mesoporous silica nanoparticles (∼100–200 nm) exhibited nondisruptive adsorption to erythrocyte surfaces; larger mesoporous silica nanoparticles (∼600 nm) induced significant local membrane deformation.^47^ Depending on the degree of hemolysis, materials can be classified into three different categories: materials resulting in over 5%, between 2 and 5%, and below 2% hemolysis are classified as hemolytic, slightly hemolytic, and nonhemolytic, respectively.^49^

Unlike other medical devices intended for systemic administration into the bloodstream, 20G-NAM will be locally implanted into bony extraction sockets. Upon implantation, the nanoparticles inevitably come into contact with red blood cells within a loose network of fibrin and other blood components. Hemolysis can release hemoglobin and heme, which may contribute to chronic inflammation and delayed tissue healing.^50^ Free heme was observed to elicit a proinflammatory response, driving macrophage polarization toward the M1 phenotype in both in vitro and in vivo models.^51^ In addition, free hemoglobin acts as a proinflammatory mediator, stimulating monocytes, macrophages, and endothelial cells via TLR4 and ROS signaling pathways.^52^ Severe hemolysis also diminishes the oxygen-carrying capacity of blood, leading to local hypoxia and subsequent impairment of tissue healing.

In this study, a 3D clotted whole blood model was modified from a previous study^32^ to assess the hemolytic potential of 20G-NAM. Figure S6 presents a schematic diagram of a 3D clotted whole blood model, comprising a blood clot matrix composed of erythrocytes, platelets, fibrin, various leukocytes (including neutrophils and monocytes), and serum. This physiologically relevant model provides a more realistic representation of the in vivo environment than traditional in vitro assays, which use diluted blood or isolated blood cells, particularly for nanoparticles intended for systemic administration. Notably, the results in Figure 8 showed that 20G-NAM displayed negligible hemolytic activity (<0.5%), indicating excellent biocompatibility with red blood cells, even at a concentration of 2000 μg/mL.

**Figure 8:**
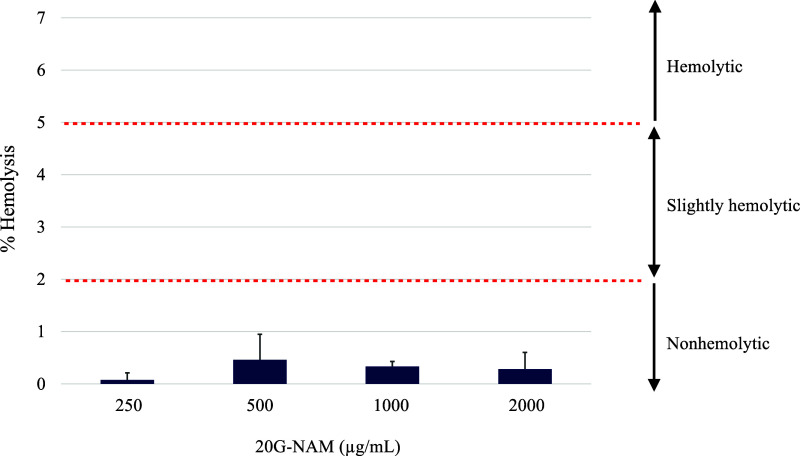
Hemolytic activity of 20G-NAM. Percent hemolysis was measured at various concentrations of 20G-NAM to assess its biocompatibility. Data are expressed as mean percent ± SD (*n* = 3). Three distinct categories of materials (nonhemolytic, slightly hemolytic, and hemolytic) classified based on their hemolytic index are shown. Notably, 20G-NAM appeared nonhemolytic, exhibiting very low hemolytic activity, even at a concentration as high as 2000 μg/mL.

A previous study using isolated red blood cells reported that bare nonsurface functionalized MCM-41 at 100–1000 μg/mL induced slight hemolysis in a concentration-dependent manner, with 3.96% hemolysis at 1000 μg/mL of MCM-41,^53^ which is substantially higher than that of 20G-NAM in our model. The significantly low hemolytic activity of 20G-NAM might be attributed to several factors, including its nanosized particles with mesopores, surface functionalization with amine and aluminum, and the barrier protection provided by clotted blood components. Mesoporous silica nanoparticles exhibit lower hemolytic activity compared with their nonporous counterparts of similar size and microsized silica particles with a similar porous structure.^54^ In addition, amine modification helps reduce the hemolysis of silica dioxide particles by blocking the surface silanols of the particles and thus diminishing the accessibility of silanol groups to the red blood cell membrane.^47,48^ Aluminum substitution reduces the concentration of surface silanol groups on MCM-41 particles.^55^ The protective effect of serum within the 3D clotted blood model against silica nanoparticle-induced hemolysis may primarily be attributed to a protein/lipid layer that shields the silica particle surface.^54^ Although GGOH exhibits high hemolytic activity of 26% at a concentration of 1725 μM,^56^ the hemolysis induced by 20G-NAM remained extremely low. The concentration of GGOH released from 20G-NAM (at 2000 μg/mL) during the 3-h hemolysis assay may be estimated from Table 2 to be approximately 20 μM, which is much lower than the reported concentration inducing hemolysis. Additionally, the fibrin network of the blood clot may help sequester released GGOH, thereby reducing its exposure to red blood cells, as previously reported.^9^ The nonhemolytic activity of 20G-NAM may also support the maintenance of the blood clot in the area of biomaterial implantation, which is essential for forming new tissue.^32,33^ By utilizing this model, future studies can also be carried out for comprehensive hemocompatibility testing of the nanoparticles, including platelet activation, coagulation, complement activation, hematology, and inflammatory/immunological responses.^57^

The dual-functional approach introduced in the present study, utilizing a nanoparticle delivery of GGOH coupled with its high ZA adsorption efficiency, represents a novel preventive strategy for MRONJ. Adsorbing ZA is critical to reduce its bioavailability to the target cells. A high ZA concentration would require a proportionally high GGOH dose for reversal, and GGOH may not fully prevent cytotoxicity induced by high ZA concentration.^7,11^ However, this high GGOH dose could be cytotoxic to the highly sensitive cells of osteoclastic lineage, especially in the absence of ZA. Therefore, reducing the initial ZA concentration enables the use of a narrow, noncytotoxic range of GGOH that is effective against the adverse effects of ZA on cells of the osteoclastic lineage. Previous studies have shown the functional benefits of bisphosphonate adsorption by calcium phosphate ceramics^5,6^ and MgO-modified porous carbon.^4^ The aluminum-modified MCM-41 nanoparticles used in this study possess a high surface area with Al^3+^ sites for ZA binding, resulting in a high ZA adsorption efficiency. Although amine functionalization with GGOH loading reduced the ZA adsorption efficiency of 20G-NAM, it remains markedly higher than that of bony inorganic hydroxyapatite. This suggests a great potential to prevent the reincorporation of free ZA into the surrounding bone. The high surface area of Al-MCM-41 also enables amine incorporation, allowing for the simultaneous delivery of GGOH at an effective concentration against ZA-induced cytotoxicity. The dual action of the resulting nanomaterial may help reduce the therapeutic dose of the material when applied locally to the bony extraction site, thus minimizing possible systemic adverse reactions. The bifunctionality of the GGOH delivery system clearly provides advantages over previously reported approaches that involve incorporating GGOH into biocompatible carriers, such as composite hydrogels,^8,9^ collagen membranes^58^, and calcium phosphate bone cements.^59^

Local delivery and biodistribution of the presently developed nanoparticles are crucial for their safety and effectiveness. To mitigate potential systemic off-targeting, an injectable hydrogel is proposed as a primary strategy for local delivery. The nanoparticles can be encapsulated in a biocompatible, injectable, and biodegradable hydrogel. While minimizing systemic exposure, the placement of encapsulated nanoparticles at the surgical site enables a high local concentration of the therapeutic agent, thereby decreasing the risk of developing MRONJ. For biodistribution studies, an In Vivo Imaging System (IVIS) can be used for nanoparticle tracking. This technique helps quantitatively assess the localized delivery and minimal systemic dissemination of the nanoparticles in future in vivo work.

## 4 Conclusions

This study presents the successful development of dual-functional NAM designed to simultaneously deliver GGOH and adsorb ZA for the prevention of MRONJ. The NAM nanoparticles demonstrated significant GGOH loading efficiency and sustained GGOH release profiles over 10 days with the release mechanism governed predominantly by Fickian diffusion as supported by Higuchi kinetic modeling. Furthermore, it could still retain moderate ZA adsorption efficiency. The results of the in vitro cytotoxicity assessments using monocy-like cells revealed that the 20G-NAM nanocarrier possessed cytocompatibility, exhibited a complete cytoprotective effect against ZA-induced impaired metabolic activity and apoptosis, and displayed negligible hemolytic activity (<0.5%).

## Supplementary Material



## Notes

X-ray diffraction pattern of aluminum-doped MCM-41 mesoporous silica nanomaterial (AM) and amine-functionalized aluminum-containing silica nanomaterial (NAM); BET surface areas, pore volumes, pore sizes, particle size distributions and mean diameters of AM and NAM; particle size distributions and mean diameters of Al-MCM-41 and N-Al-MCM-41 nanoparticles; overlaid TGA curves of the GGOH-loaded nanomaterials and their GGOH-free starting nanomaterials; linear regression coefficients (*R* ^2^) and drug release rate constant (*k*) values obtained after fitting the GGOH release profiles of the GGOH-loaded nanomaterials; representative differential interference contrast (DIC) image of multinucleated giant cells within multicellular clusters of cells treated with AM; various-sized 20G-NAM aggregates in 4-h cultured RAW cells treated with 20G-NAM; representative flow cytometric FSC-FITC dot plots showing the cellular uptake of autofluorescent 20G-NAM nanoparticles by RAW cells; and a schematic diagram of a 3D clotted whole blood model for evaluating the hemolytic activity of 20G-NAM (PDF)
