# Supplementary material for: Dual-Functional Amine-Modified Aluminum-Doped MCM-41 Nanoparticles for Concurrent Zoledronic Acid Adsorption and Geranylgeraniol Delivery for Prevention of Medication-Related Osteonecrosis of the Jaw
Source: ACS Mater Au. 2025 Oct 13;5(6):1037–51. doi: 10.1021/acsmaterialsau.5c00112 (PMC12616448; doi:10.1021/acsmaterialsau.5c00112)
Supplement: Supplementary file 1 [file mg5c00112_si_001.pdf]

# Supporting Information

## Dual-Functional Amine-Modified Aluminum-Doped MCM-41 Nanoparticles for Concurrent Zoledronic Acid Adsorption and Geranylgeraniol Delivery for Prevention of Medication-Related Osteonecrosis of the Jaw

Pornchanok Pichaipanich[1], Weerachai Singhatanadgit[2*], Boonlom Thavornyutikarn[1], Piyarat Sungkhaphan[1], Setthawut Kitpakornsanti[2], Soraya Pornsuwan[3], and Wanida Janvikul[1*]

[1]National Metal and Materials Technology Center, National Science and Technology Development Agency, Pathum-thani, 12120, Thailand

[2]Faculty of Dentistry and Research Unit in Mineralized Tissue Reconstruction, Thammasat University (Rangsit Campus), Pathum-thani, 12121, Thailand

[3]Faculty of Science, Mahidol University, Bangkok, 10400, Thailand

*Authors for correspondence

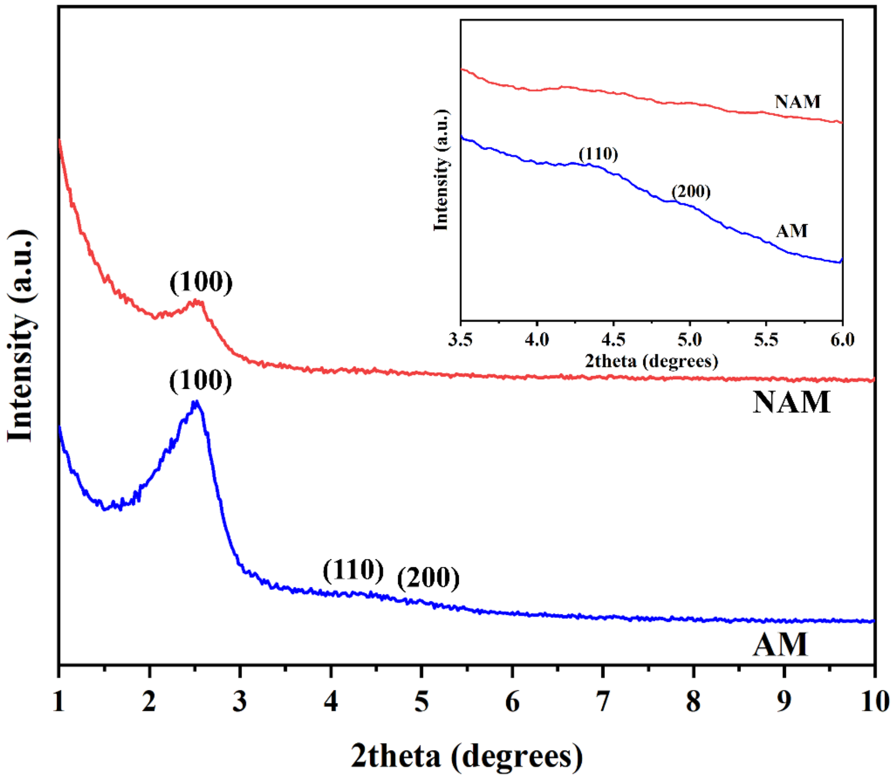


**Figure S1.** The X-ray diffraction patterns of aluminum-doped mesoporous silica nanomaterial (AM) and amine-functionalized aluminum-containing silica nanomaterial (NAM). The inset shows the magnified view of the diffraction peaks corresponding to the (110) and (200) reflections.

**Table S1.** Summary of BET surface areas, pore volumes, and pore sizes of AM and amine-functionalized AM (NAM)

| Particle characteristic | Al-MCM-41 | N-Al-MCM-41 |
|---|---|---|
| Surface area ($m^2/g$) | 752.8 | 115.16 |
| Pore volume ($cm^3/g$) | 0.73 | 0.16 |
| Pore size (Å) | 23.55 | 54.04 |

**Table S2.** Particle size distributions and mean diameters of Al-MCM-41 and N-Al-MCM-41 nanoparticles

| Sample | Value of specific percentile (μm) | | |
|---|---|---|---|
| | P10 | P50 | P90 |
| Al-MCM-41 | 15.58 ± 0.32 | 154.89 ± 1.77 | 410.22 ± 2.30 |
| N-Al-MCM-41 | 8.74 ± 0.04 | 66.18 ± 1.26 | 172.01 ± 0.92 |

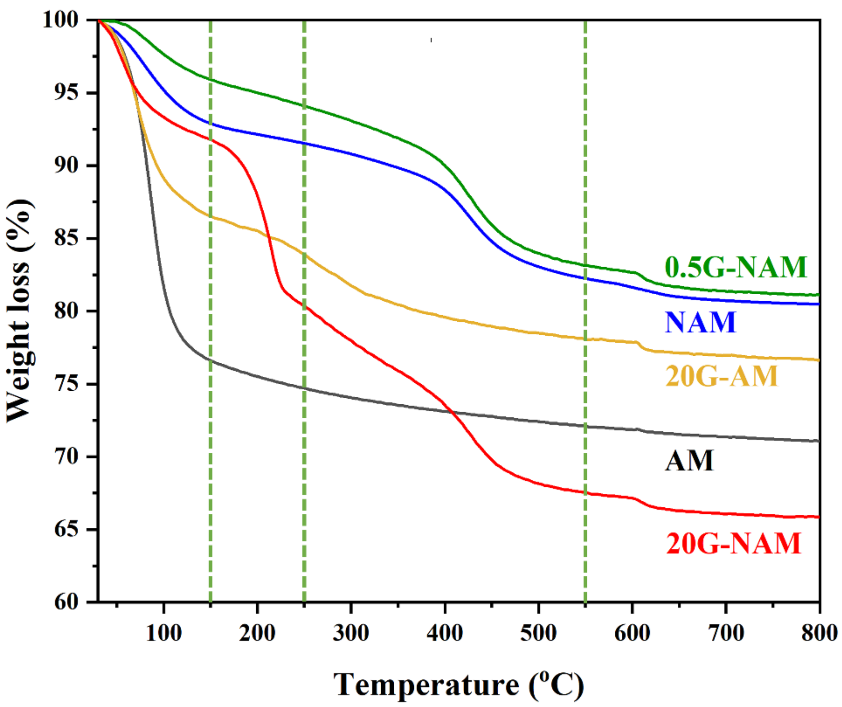


**Figure S2.** Overlaid TGA curves of the GGOH-loaded nanomaterials and their GGOH-free starting nanomaterials.

**Table S3.** Linear regression coefficients ($R^2$) and drug release rate constant (k) values obtained after fitting the GGOH release profiles of the GGOH-loaded nanomaterials.

| Sample | First-order | | Higuchi | | Korsmeyer-Pappas | | Hixson-Crowell | |
|---|---|---|---|---|---|---|---|---|
| | k | $R^2$ | k | $R^2$ | k | $R^2$ | k | $R^2$ |
| 20G-AM | 0.0173 | 0.9899 | 6.5873 | 0.9551 | 1.2053 | 0.9960 | -0.1488 | 0.9668 |
| 0.5G-NAM | 0.0177 | 0.9822 | 6.8245 | 0.9988 | 3.1074 | 0.9909 | -0.1182 | 0.9032 |
| 20G-NAM | 0.1009 | 0.9856 | 22.8600 | 0.9868 | 18.0219 | 0.9731 | -0.1475 | 0.8710 |

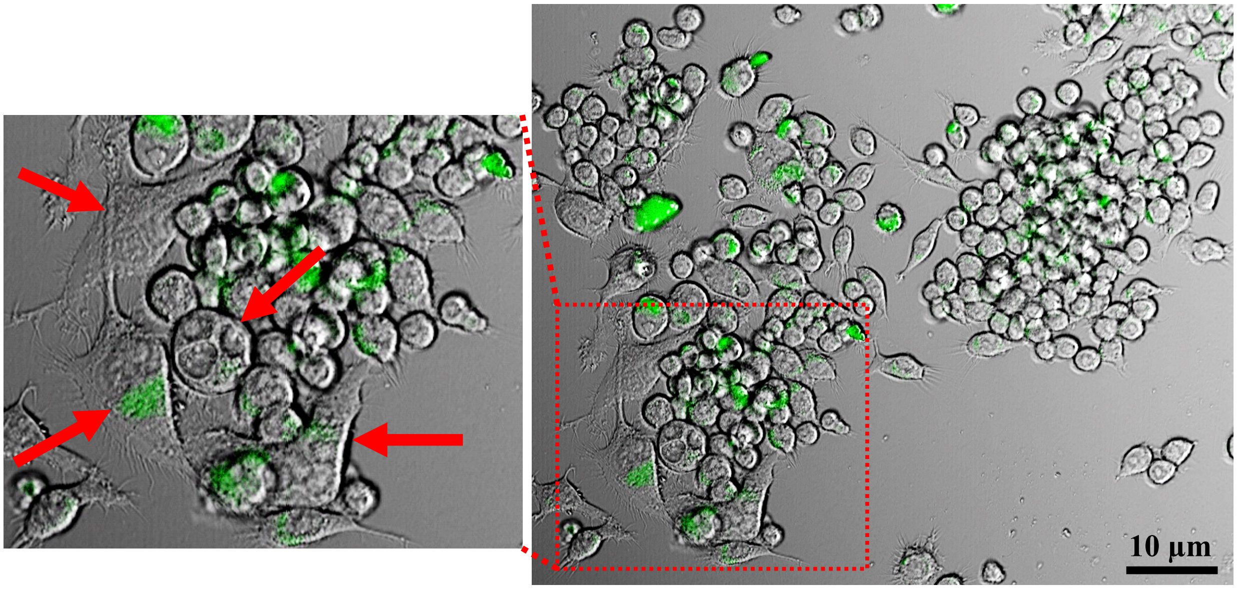


**Figure S3.** A representative differential interference contrast (DIC) image of multinucleated giant cells within multicellular clusters of 24-h cultured RAW cells after 2-h treatment with 300 µg/mL AM nanoparticles, visualized under a confocal fluorescence microscope. Green fluorescence indicates the autofluorescence of AM nanoparticles/aggregates associated with cells. Red arrows indicate multinucleated giant cells.

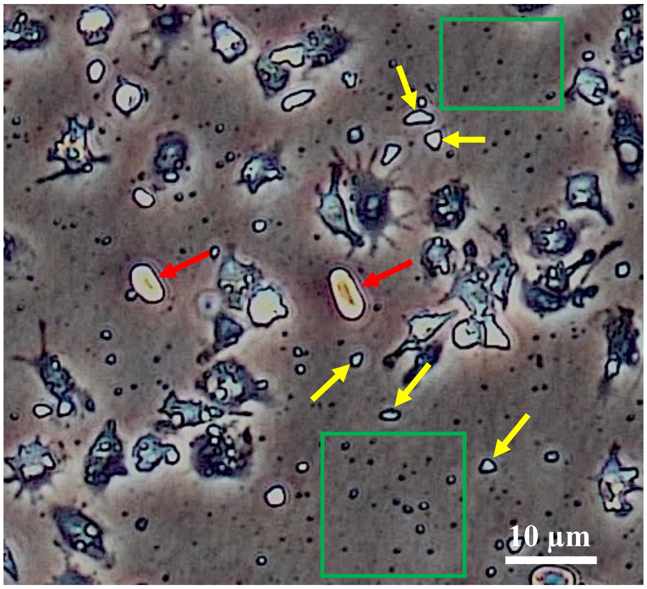


**Figure S4.** Various-sized 20G-NAM aggregates in 4-h cultured RAW cells treated with 300 µg/mL 20G-NAM, visualized under a phase-contrast microscope. Aggregates smaller than 1 µm, 1-3 µm, and larger than 3 µm are indicated by green squares, yellow arrows, and red arrows, respectively.

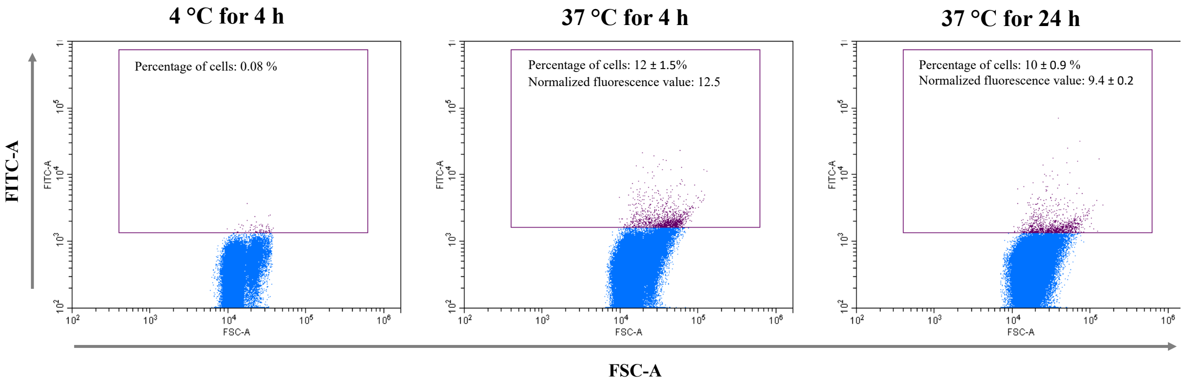


**Figure S5**. Representative flow cytometric FSC-FITC dot plots showing the cellular uptake of autofluorescent 20G-NAM nanoparticles by RAW cells. Cells were treated with 20G-NAM (200 μg/mL) at 4 °C or 37 °C for 4 or 24 h. The percentage of cells with a fluorescence signal above the cellular background signal (indicated by the blue dots) is shown within the purple boxes. These cells were considered to have internalized the 20G-NAM nanoparticles. The normalized fluorescence value, which is proportional to the 20G-NAM uptake level, was calculated by dividing the geometric mean of the FITC signal (above the cellular background) by the geometric mean of the cellular background. Both the percentages of cells that internalized 20G-NAM and the normalized fluorescence values are displayed in the dot plots. Data are expressed as the mean ± SD (n = 3). It was noted that only a small proportion of cells (12 %) could uptake 20G-NAM by an energy-dependent internalization pathway, not by free diffusion which can occur at 4 °C.

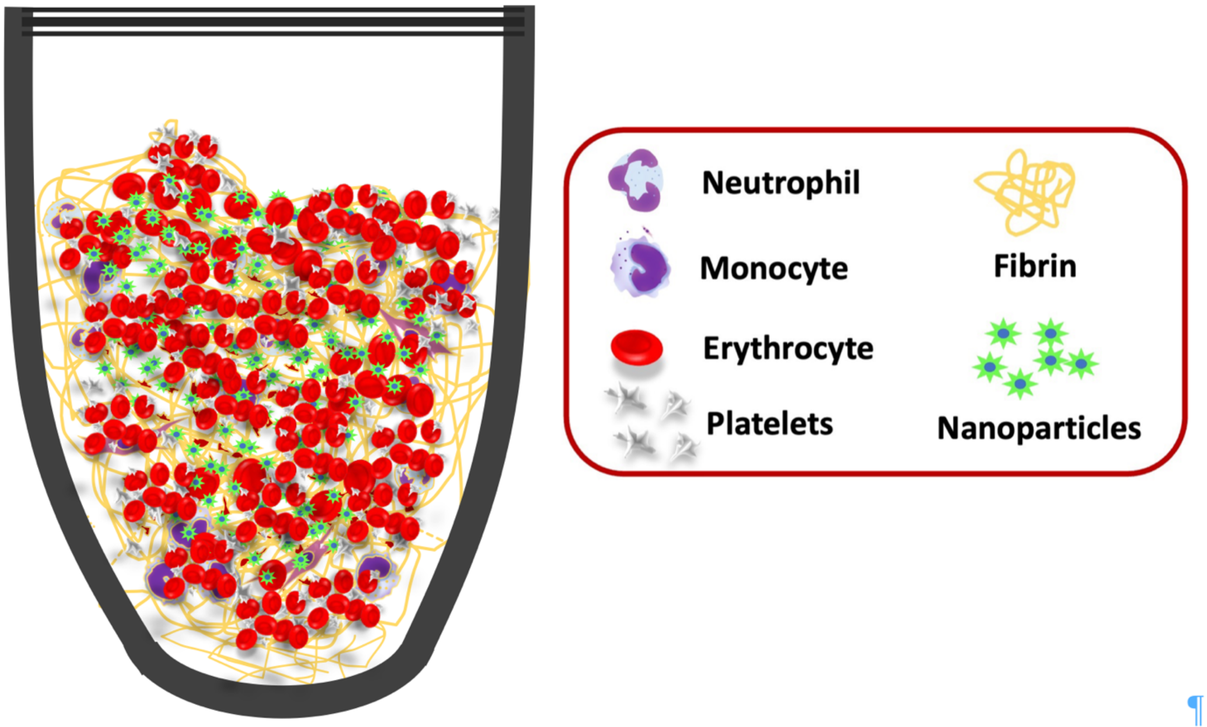


¶

**Figure S6.** A schematic diagram of a 3D clotted whole blood model for evaluating the hemolytic activity of 20G-NAM. The model consists of a clotted blood matrix, composed of erythrocytes, platelets, fibrin, various white blood cells (e.g., neutrophils and monocytes), and serum fluid. The nanoparticles (represented by green stars) are dispersed within the clotted blood, allowing for the investigation of their interactions with red blood cells in a physiologically relevant microenvironment.
